# Susceptibility Loci Associated with Specific and Shared Subtypes of Lymphoid Malignancies

**DOI:** 10.1371/journal.pgen.1003220

**Published:** 2013-01-17

**Authors:** Joseph Vijai, Tomas Kirchhoff, Kasmintan A. Schrader, Jennifer Brown, Ana Virginia Dutra-Clarke, Christopher Manschreck, Nichole Hansen, Rohini Rau-Murthy, Kara Sarrel, Jennifer Przybylo, Sohela Shah, Srujana Cheguri, Zsofia Stadler, Liying Zhang, Ora Paltiel, Dina Ben-Yehuda, Agnes Viale, Carol Portlock, David Straus, Steven M. Lipkin, Mortimer Lacher, Mark Robson, Robert J. Klein, Andrew Zelenetz, Kenneth Offit

**Affiliations:** 1 Clinical Genetics Service, Department of Medicine, Memorial Sloan-Kettering Cancer Center, New York, New York, United States of America; 2 Cancer Biology and Genetics Program, Sloan-Kettering Institute, New York, New York, United States of America; 3 New York University Cancer Institute, New York University School of Medicine, New York, New York, United States of America; 4 Dana Farber Cancer Institute, Boston, Massachusetts, United States of America; 5 Diagnostic Molecular Genetics Laboratory, Department of Pathology, Memorial Sloan-Kettering Cancer Center, New York, New York, United States of America; 6 Department of Hematology, Hadassah-Hebrew University Medical Center, Jerusalem, Israel; 7 Genomics Core, Memorial Sloan-Kettering Cancer Center, New York, New York, United States of America; 8 Lymphoma Service, Department of Medicine, Memorial Sloan-Kettering Cancer Center, New York, New York, United States of America; 9 Weill Cornell Medical Center, New York, New York, United States of America; Cincinnati Children's Hospital Medical Center, United States of America

## Abstract

The genetics of lymphoma susceptibility reflect the marked heterogeneity of diseases that comprise this broad phenotype. However, multiple subtypes of lymphoma are observed in some families, suggesting shared pathways of genetic predisposition to these pathologically distinct entities. Using a two-stage GWAS, we tested 530,583 SNPs in 944 cases of lymphoma, including 282 familial cases, and 4,044 public shared controls, followed by genotyping of 50 SNPs in 1,245 cases and 2,596 controls. A novel region on 11q12.1 showed association with combined lymphoma (LYM) subtypes. SNPs in this region included rs12289961 near *LPXN*, (P_LYM_ = 3.89×10^−8^, OR = 1.29) and rs948562 (P_LYM_ = 5.85×10^−7^, OR = 1.29). A SNP in a novel non-HLA region on 6p23 (rs707824, P_NHL_ = 5.72×10^−7^) was suggestive of an association conferring susceptibility to lymphoma. Four SNPs, all in a previously reported HLA region, 6p21.32, showed genome-wide significant associations with follicular lymphoma. The most significant association with follicular lymphoma was for rs4530903 (P_FL_ = 2.69×10^−12^, OR = 1.93). Three novel SNPs near the HLA locus, rs9268853, rs2647046, and rs2621416, demonstrated additional variation contributing toward genetic susceptibility to FL associated with this region. Genes implicated by GWAS were also found to be cis-eQTLs in lymphoblastoid cell lines; candidate genes in these regions have been implicated in hematopoiesis and immune function. These results, showing novel susceptibility regions and allelic heterogeneity, point to the existence of pathways of susceptibility to both shared as well as specific subtypes of lymphoid malignancy.

## Author Summary

B-cell lymphomas comprise several diseases representing aberrant proliferations of immune cells at various stages of maturation. It might be expected that dissimilar subtypes of lymphoma will have different etiologic and pathogenic mechanisms, reflecting the distinct histologic and clinical characteristics of these diseases. This study aims to define both shared as well as specific genetic risk factors for lymphoma. Utilizing a genome-wide approach, we discovered novel locations in the genome associated with risk for lymphoid malignancies. Common variants in these regions, on chromosome 11q12.1 and 6p23, were each associated with a modest modification of risk for lymphoma. These regions harbor several genes of biological importance in lymphoid maturation and function. We also further characterized the HLA region at 6p21.32, previously associated with lymphoma risk and thought to be important in immune function. Some of the associated SNP markers were specific for one common subtype of lymphoma, e.g. follicular lymphoma. However, others were associated with combined subsets of disease, suggesting that there are both shared and subtype-specific associations between common genetic variants and human lymphoid cancer. Secondary analyses showed that the two novel regions harbor candidates that are biologically relevant and that regulate cell development and hematopoiesis.

## Introduction

Lymphoid malignancies represent clonal proliferations occurring at various stages of differentiation of B and T cells. B-cell differentiation is characterized by a canonical set of DNA modifications, including somatic hypermutation, class switching, and VDJ recombination. If aberrant, these result in lymphoid neoplasms ranging from less differentiated acute leukemia and lymphoma, to well-differentiated plasma cell malignancies [1]. Some genetic and environmental risk factors for lymphoma have been defined and antecedent autoimmune disorders increase risk for lymphoma several fold [2]. Familial clustering of lymphomas has been observed and may comprise mixed phenotypes of Hodgkin's lymphoma (HD) as well as the subsets of non-Hodgkin's (NHL) including follicular (FL), diffuse large B-cell (DLBCL), and chronic lymphocytic/small lymphocytic (CLL/SLL) [3]. While less common than B cell neoplasms, T cell malignancies are also part of the spectrum of familial lymphoma and may be seen alone or in combination with B cell neoplasms in kindreds with underlying immune deficiency or genomic instability [3].

The lack of genetic linkage to specific loci in such families has prompted the search for common susceptibility variants in the germline, which may provide evidence as to the etiology of these disorders. Genome wide association studies (GWAS) examining lymphoma susceptibility have focused on identifying risk loci associated with different subtypes of the disease, based on the *a priori* assumption that each of the subtypes have distinct biology and therefore, distinct pathogenesis. Thus far, a locus on 6p21.33, near *PSOR1*, and another region at 6p21.32, near *HLA-DRB1* have been associated with FL [4], [5], [6] and Hodgkin's disease [7], [8]. A smaller study has described *CDC42BPB* at 14q32 to be associated with diffuse large cell lymphoma [9].

In order to test the paradigm that there are common and subtype specific germline susceptibility loci for lymphoma, we conducted a two-stage genome-wide association study (GWAS). Our stage-1 consisted of 944 cases of lymphoma, including 282 familial cases, and 4044 public shared controls. Stage-2 consisted of 1245 cases and 2596 controls. We have used a higher ratio of controls to cases to enhance power to detect association, as the use of public shared controls comes at no cost [10]. We also analyzed published data for overlap of the GWAS hits to expression quantitative trait loci (eQTL) in lymphoblastoid cell lines. Secondary analyses, such as gene set enrichment were carried out to detect enrichment of biologically relevant candidates for further study.

## Results

The study design consisted of two phases, Stage-1 comprising the GWAS of lymphoma and shared controls and Stage-2 comprising 50 SNPS selected from the Stage-1 for replication.

### Stage-1 results

**Figure 1:**
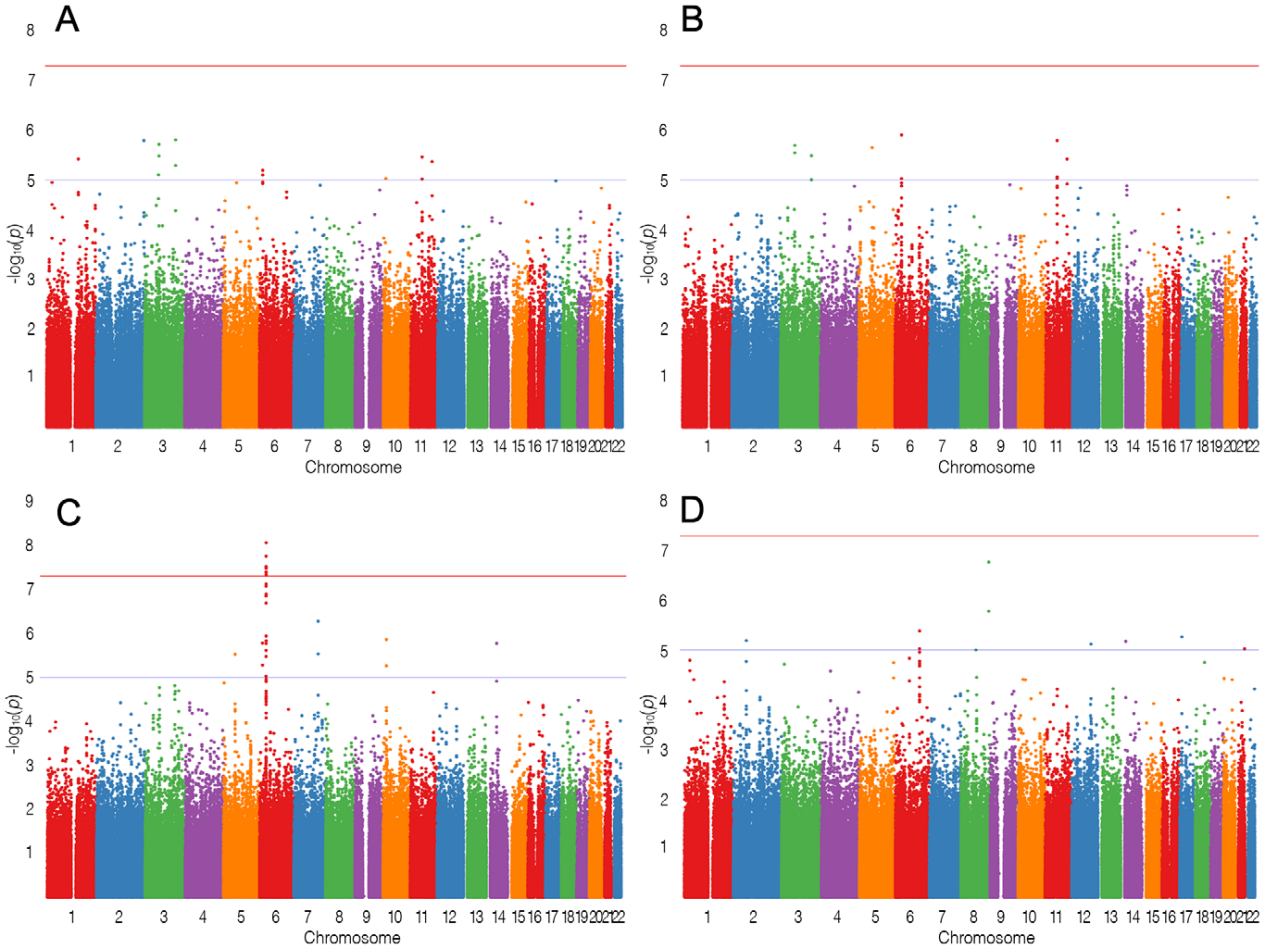
Manhattan plots. Manhattan plots for (A) LYM, (B) NHL, (C) FL, (D) DLBCL. The blue line shows suggestive association and the red line genome-wide association. X-axis labels correspond to chromosomes, Y-axis shows −log_10_(P) from logistic regression.

**Figure 2:**
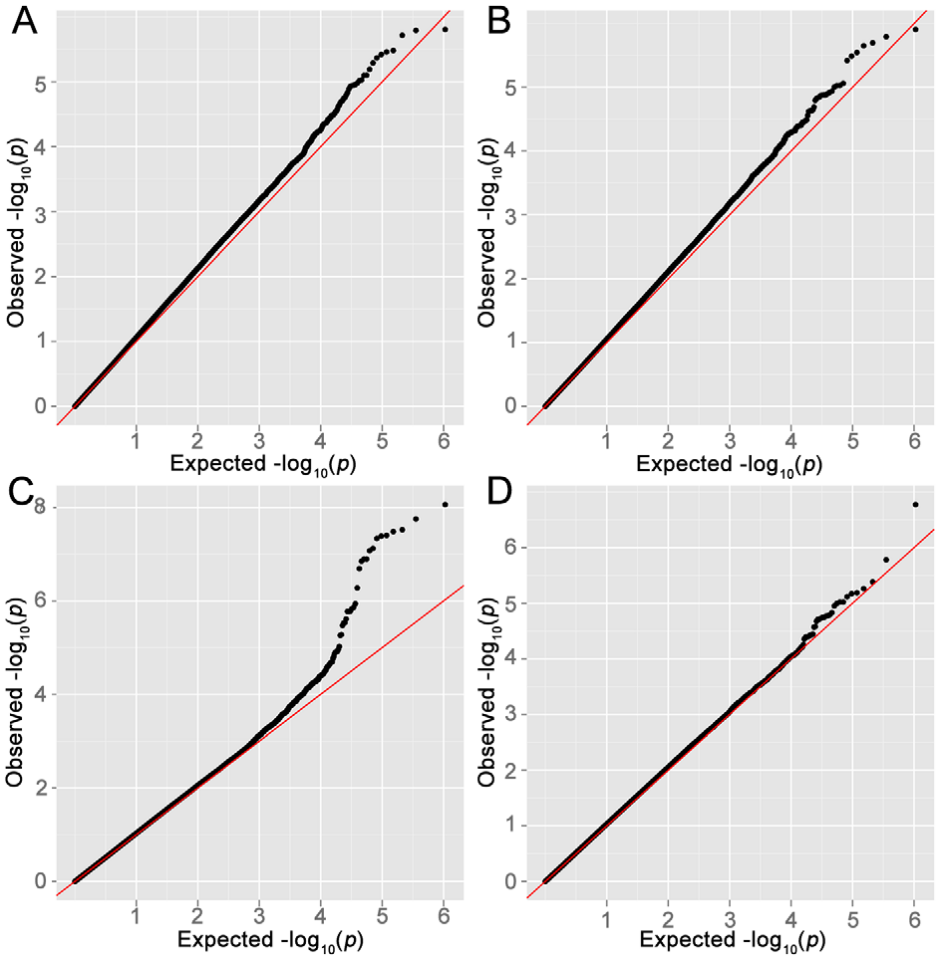
Quantile–quantile plots. Quantile–quantile (QQ) plots for (A) LYM, (B) NHL, (C) FL, (D) DLBCL. The X-axis represents expected −log_10_(P) and the Y-axis represents −log_10_(P) from logistic regression. Genomic inflation factor λ for LYM, NHL, FL and DLBCL was 1.09, 1.07, 1.04 and 1.04 respectively.

In stage-1, we analyzed 944 cases of lymphoma, including 275 FL, and 4044 controls and documented strong evidence of association between SNPs on Chr6, with at least 9 SNPs showing P_FL_<1×10^−7^ at the HLA region (chr6:32.17–32.89 Mb) encompassing genes *TNXB* to *HLA-DOB*. The results of the stage-1 analysis for LYM, NHL, FL and DLBCL are shown as Manhattan plots (Figure 1) and quantile-quantile (QQ)-plots (Figure 2). FL showed the strongest enrichment of association signals; particularly on Chr6. We refrained from detailed analysis of smaller subsets, based on the power calculations performed using PGA [11] taking into account sample sizes, detectable relative risk and case to control ratios (Figure S1).Analysis of the major classifiers LYM and NHL and only the major subgroups FL, DLBCL were performed. In addition, a subset designated as NFD comprised any non-Hodgkin's lymphoma cases that were neither FL nor DLBCL. This subgroup was created to test if the associations in the larger LYM and NHL were driven primarily by the pre-dominant subgroups FL and DLBCL.

**Figure 3:**
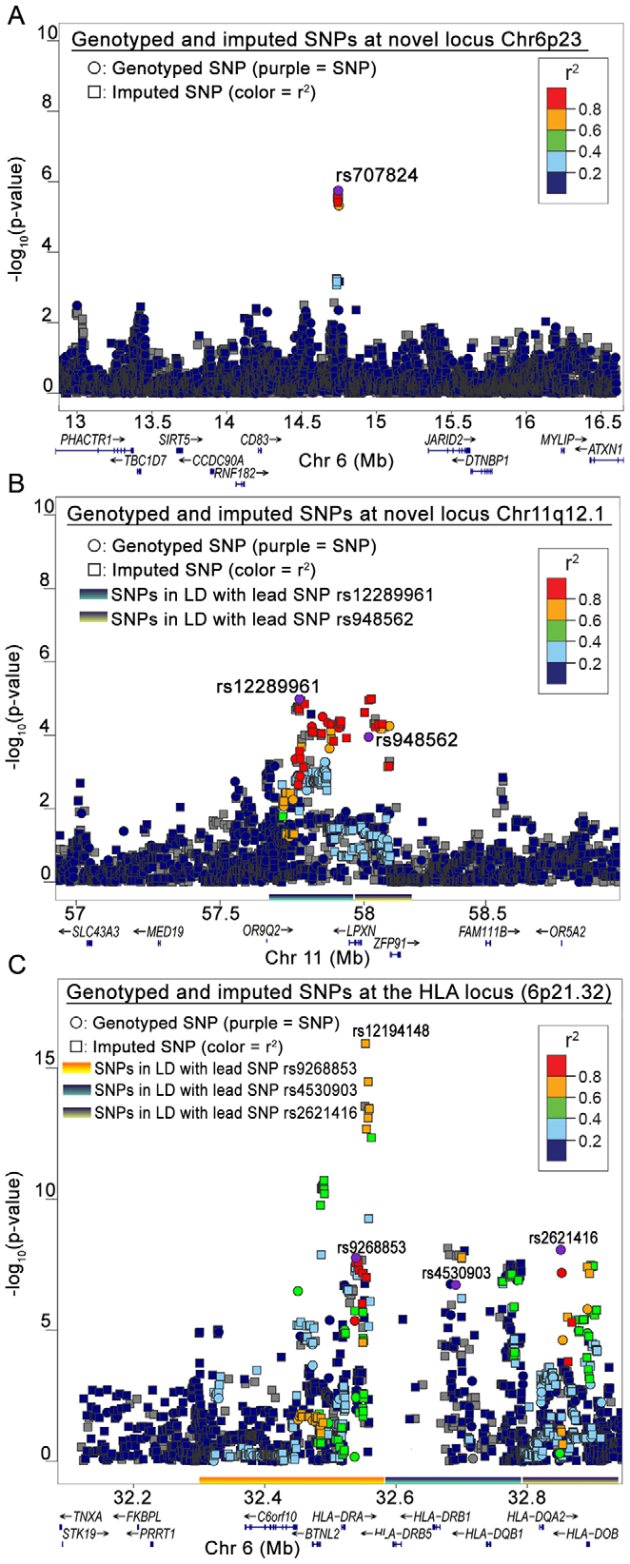
Regional plots from stage-1 GWAS. (A) Chr6p23, (B) Chr11q12.1 and (C) Chr 6p21.32. The regions corresponding to LD between lead SNPs and other SNPs are marked on the X axis of the plot.

Among all analyses, the lowest p-values in the FL subset were observed on chromosome 6p. The smallest p-value was for rs2621416 (P_FL_ = 8.69×10^−9^, OR 1.82) (Table S1) followed by rs9268853 (P_FL_ = 1.76×10^−8^, OR = 1.74).Imputation of the stage-1 data revealed strong associations with FL for the 6p21.32 SNP rs12194148 (P_FL_ = 1.18×10^−16^, 14.5 kb from rs9268853; r^2^ = 0.62, D′ = 1.0), suggesting a subtype specific association with the HLA locus (Figure 3C). In addition to the SNPs on chromosome 6p HLA region, we also found preliminary evidence of association of several SNPs at chromosome 3q25.2 with LYM, NHL and NFD. Another locus at 11q12.1 was defined by two SNPs with suggestive associations (P<10^−5^) (Table S1, Figure 3B).

### Stage-2 results

Fifty SNPs were selected from stage 1 for genotyping in a larger set of 1245 lymphomas (Table S1). After adjusting for age and Jewish ancestry, nine of 50 SNPs had P-values below the nominal alpha level of 0.05, while showing the same direction of effect as observed in stage 1 (Table S2). After adjusting for the 50 SNPs tested, rs4530903, at the HLA locus, remained significantly associated with NHL, FL, and DLCBCL. This SNP also appears to be associated with LYM, but the p-value was marginally higher than the Bonferroni corrected threshold. Two other tests were significant after multiple test correction: rs707824 on chromosome 6p23 with NHL and rs12289961 on chromosome 11q12.1 with LYM. Thus, two novel susceptibility loci replicated in stage 2. Notably, the SNPs at 11q12.1 also are nominally significant (P<0.05) in the NFD subgroup, which is different from the observation for the SNPs at 6p21.32. Based on this analysis, nine of these SNPs were advanced to a meta-analysis of both stage-1 and stage-2 data (Table 1).

**Table 1:** Summary statistics for the analyses in all three stages.

	SNP	rs707824	rs9268853	rs4530903	rs2647046	rs9276490	rs7453920	rs2621416	rs12289961	rs948562
SNP Details	Locus	6p23	6p21.32	6p21.32	6p21.32	6p21.32	6p21.32	6p21.32	11q12.1	11q12.1
	RA	T	C	T	A	A	A	G	T	G
	Genes Nearby	RNF182, JARID2	nongenic	HLA-DRB5, HLA-DQA1	HLA-DQB1, HLA-DQA2	HLA-DQA2, HLA-DQB2	HLA-DQB2	HLA-DQB2, TAP2	nongenic	ZFP91-CNTF
		9.13×10^−3^	3.7×10^−2^	4×10^−3^	4×10^−3^	4.29×10^−4^	3.96×10^−4^	1.08×10^−3^	9.63×10^−6^	5.72×10^−5^
	**LYM**	1.26	1.14	1.29	0.83	0.8	0.78	1.24	1.37	1.35
		(1.06–1.50)	(1.01–1.48)	(1.09–1.53)	(0.74–0.94)	(0.71–0.91)	(0.71–0.91)	(1.09–1.41)	(1.19–1.57)	(1.17–1.57)
		7.98×10^−4^	4.5×10^−4^	9.42×10^−6^	1.33×10^−5^	2.65×10^−4^	3.27×10^−4^	1.24×10^−6^	9.36×10^−6^	8.70×10^−6^
	**NHL**	1.39	1.27	1.51	0.73	0.78	0.78	1.42	1.42	1.45
		(1.15–1.68)	(1.11–1.45)	(1.26–1.82)	(0.64–0.84)	(0.68–0.89)	(0.68–0.89)	(1.23–1.64)	(1.21–1.65)	(1.32–1.70)
		1.67×10^−6^	1.76×10^−8^	3.29×10^−8^	3.97×10^−8^	8.33×10^−4^	6.8×10^−4^	8.69×10^−9^	1.1×10^−3^	4.0×10^−3^
**Discovery Stage**	**FL**	1.89	1.74	2.01	0.53	0.7	0.69	1.82	1.45	1.41
**(944 Cases,**		(1.46–2.45)	(1.43–2.10)	(1.57–2.57)	(0.42–0.66)	(0.57–0.86)	(0.56–0.86)	(1.48–2.23)	(1.16–1.81)	(1.11–1.79)
**4044 Controls)**		8.24×10^−2^	9.84×10^−1^	3.45×10^−2^	1.77×10^−1^	4.31×10^−3^	7.05×10^−3^	3.99×10^−3^	1.47×10^−1^	1.1×10^−2^
	**DLBCL**	1.29	1	1.36	0.87	0.74	0.75	1.37	1.19	1.37
		(0.97–1.71)	(0.81–1.22)	(1.02–1.79)	(0.71–1.07)	(0.60–0.91)	(0.61–0.92)	(1.11–1.69)	(0.94–1.50)	(1.08–1.74)
		5.98×10^−1^	4.10×10^−1^	2.70×10^−1^	6.25×10^−1^	2.95×10^−1^	2.69×10^−1^	5.13×10^−1^	1.05×10^−4^	4.24×10^−3^
	**NFD**	0.93	0.93	0.86	1.04	0.91	0.91	0.93	1.45	1.34
		(0.72–1.20)	(0.78–1.11)	(0.66–1.12)	(0.88–1.23)	(0.77–1.08)	(0.77–1.08)	(0.78–1.13)	(1.20–1.74)	(1.10–1.64)
		1.78×10^−3^	4.31×10^−1^	5.79×10^−4^	5.46×10^−3^	9.28×10^−3^	4.98×10^−3^	2.75×10^−1^	8.29×10^−4^	1.33×10^−3^
	**LYM**	1.26	1.04	1.29	0.85	0.87	0.86	1.06	1.22	1.23
		(1.09–1.45)	(0.94–1.16)	(1.12–1.49)	(0.76–0.95)	(0.78–0.97)	(0.77–0.95)	(0.95–1.19)	(1.09–1.39)	(1.09–1.41)
		3.34×10^−4^	3.96×10^−1^	3.48×10^−4^	1.38×10^−2^	3.06×10^−2^	1.18×10^−2^	9.12×10^−2^	1.74×10^−3^	2.54×10^−3^
	**NHL**	1.31	1.05	1.31	0.86	0.89	0.87	1.09	1.21	1.22
		(1.13–1.50)	(0.94–1.16)	(1.13–1.51)	(0.76–0.97)	(0.80–0.99)	(0.77–0.97)	(0.98–1.24)	(1.08–1.38)	(1.07–1.40)
		1.93×10^−1^	1.24×10^−3^	1.01×10^−5^	1.2×10^−3^	9.13×10^−2^	4.35×10^−2^	1.17×10^−2^	6.75×10^−2^	3.19×10^−1^
**Replication Stage**	**FL**	1.23	1.42	1.83	0.67	0.83	0.79	1.31	1.26	1.13
**(1245 Cases,**		(0.90–1.64)	(1.15–1.75)	(1.40–2.38)	(0.52–0.85)	(0.67–1.03)	(0.64–0.99)	(1.07–1.66)	(0.98–1.60)	(0.88–1.50)
**2596 Controls)**		1.85×10^−3^	9.7×10^−1^	5.36×10^−4^	3.09×10^−1^	9.47×10^−2^	8.13×10^−2^	9.27×10^−2^	1.97×10^−2^	3.24×10^−2^
	**DLBCL**	1.41	1	1.46	0.91	0.87	0.86	1.15	1.25	1.24
		(1.14–1.76)	(0.84–1.18)	(1.18–1.82)	(0.76–1.09)	(0.73–1.03)	(0.72–1.02)	(0.98–1.39)	(1.04–1.51)	(1.02–1.53)
		1.65×10^−2^	3.16×10^−1^	7.56×10^−1^	1.21×10^−1^	6.77×10^−2^	5.24×10^−2^	5.67×10^−1^	1.64×10^−2^	1.00×10^−2^
	**NFD**	1.22	0.94	1.03	0.89	0.89	0.88	0.96	1.19	1.23
		(1.04–1.44)	(0.83–1.06)	(0.86–1.23)	(0.78–1.03)	(0.78–1.01)	(0.77–1.00)	(0.84–1.10)	(1.03–1.37)	(1.05–1.42)
		2.49×10^−5^	5.4×10^−2^	3.14×10^−6^	7.37×10^−5^	1.21×10^−5^	4.92×10^−6^	2.35×10^−3^	3.89×10^−8^	5.85×10^−7^
	**LYM**	1.26	1.08	1.29	0.84	0.84	0.84	1.14	1.29	1.29
**Combined Stage**		(1.13–1.39)	(0.99–1.16)	(1.16–1.43)	(0.77–0.92)	(0.78–0.91)	(0.77–0.90)	(1.05–1.25)	(1.17–1.40)	(1.16–1.43)
**(meta-analysis)**		5.72×10^−7^	4.25×10^−3^	1.57×10^−8^	2.35×10^−6^	1.18×10^−4^	4.14×10^−5^	1.56×10^−5^	9.98×10^−8^	2.89×10^−7^
	**NHL**	1.33	1.12	1.37	0.8	0.84	0.83	1.22	1.29	1.32
		(1.17–1.47)	(1.03–1.21)	(1.23–1.54)	(0.73–0.88)	(0.77–0.92)	(0.76–0.91)	(1.11–1.33)	(1.17–1.42)	(1.18–1.46)

Association with all lymphoma subtypes (LYM), all non-Hodgkin's lymphomas (NHL), and subtypes of follicular lymphoma (FL) and diffuse large B cell lymphoma (DLBCL) and all non-follicular or non-diffuse subtypes (NFD), RA = Risk allele.

### Meta-analysis of the combined Stage-1 and Stage-2

#### Confirmation of the 6p21.32 HLA association in FL

To combine data from stages 1 and 2, a meta-analysis of nine SNPs for one or more subtypes of lymphoma (Table 1) was performed. We replicated the previously reported association with 6p21.32 region and FL; a novel SNP in this region, rs4530903 was associated with both FL (P_FL_ = 2.69×10^−12^, OR = 1.93) as well as NHL (P_NHL_ = 1.57×10^−8^, OR = 1.37). rs4530903 was correlated with a previously reported SNP, rs10484561 (r^2^ = 0.84, D′ = 0.95), associated with FL [6]. In addition, rs9268853 (P_FL_ = 2.48×10^−10^, OR = 1.56) and rs2647046 (P_FL_ = 3.77×10^−10^, OR = 0.59) were also significantly associated with FL (r^2^ = 0.43, D′ = 1.0). These SNPs showed very little evidence of association in DLBCL. Three of these nine SNPs mapped to two chromosomal regions not previously reported, 6p23 and 11q12.1. The SNP at 6p23, rs707824 (P_NHL_ = 5.72×10^−7^, OR = 1.33) fell just below the genome wide threshold of significance.

#### Two novel SNPs associated with LYM and NHL at 11q12.1

Two SNPs in a novel region at 11q12.1 that were significantly associated with combined lymphoma subtypes were discovered. rs12289961, a nongenic SNP, showed evidence of association in the combined meta-analysis of the two phases (P_LYM_ = 3.89×10^−8^, OR = 1.29). Another SNP, rs948562 at 11q12.1, 287 kb distal to rs12289961, showed a similar trend for association (P_LYM_ = 5.85×10^−7^, OR = 1.29; P_NHL_ = 2.89×10^−7^, OR = 1.32). These SNPs are weakly correlated (r^2^ = 0.6, D′ = 0.86) and were not highly significant for the major subtypes FL and DLBCL (Table 1). However, these SNPs were the most significant of the nine SNPs in the NFD group, suggesting that the FL and DLBCL signals were not driving these associations. Heterogeneity amongst the major subtypes FL, DLBCL, and NFD was not seen for the SNPs on chr11q12.1 and 6p23, while all SNPs except rF453920 at 6p21.32 showed heterogeneity in effect sizes (Table 2). The same trend was found when these SNPs were tested amongst cases with and without a family history of lymphoma (Table 2). These data suggest evidence of a novel mechanism of shared susceptibility to lymphoma associated with the 11q12.1 and 6p23 regions. The evidence for heterogeneity at 6p21.32 was weaker when HD was excluded from the analysis.

**Table 2:** Heterogeneity test.

	CHR	SNP	P (BD)
**Heterogeneity test for SNPs in FL, DLBCL, and NFD**	6	rs707824	0.32
	6	rs9268853	4.64×10^−8^
	6	rs4530903	6.81×10^−8^
	6	rs2647046	0.002
	6	rs9276490	0.04
	6	rs7453920	0.14
	6	rs2621416	3.75×10^−6^
	11	rs12289961	0.44
	11	rs948562	0.66
**Heterogeneity within familial and sporadic lymphoma**	6	rs707824	0.88
	6	rs9268853	0.28
	6	rs4530903	0.01
	6	rs2647046	1.98×10^−4^
	6	rs9276490	1.17×10^−4^
	6	rs7453920	3.13×10^−4^
	6	rs2621416	6.30×10^−4^
	11	rs12289961	0.88
	11	rs948562	0.60

(P) BD is the asymptotic p-value of Breslow-Day statistic for the heterogeneity test.

**Figure 4:**
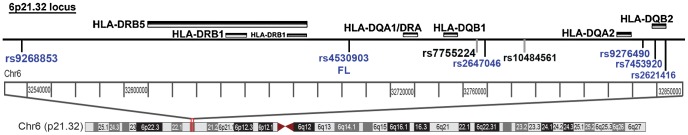
The Chr6p21.32 locus depicting the novel loci near the HLA II locus. SNPs marked in blue are novel SNPs that are significant in LYM, NHL or FL. The two SNPs previously reported are rs7755224 and rs10484561 (in black).

The data show the known regions of association with FL at 6p21.32 to include four more SNPs. These novel SNPs were rs2647046 (P_FL_ = 3.77×10^−10^, OR = 0.59), rs9268853 (P_FL_ = 2.48×10^−10^, OR = 1.56), and rs2621416 (P_FL_ = 2.41×10^−9^, OR = 1.57, Figure 4). Two of these SNPs at 6p21.32, rs9268853 and rs2621416, were predominantly associated with FL and did not show an association with the aggregate set of all LYM (Table 1). SNPs rs4530903, rs707824 and rs2647046 demonstrated p-value less than 5×10^−5^ in NHL, likely driven by the association signal in FL and DLBCL at this locus. In addition, SNPs rs9268853, rs4530903 and rs2621416 showed association (P<0.05) with the HD subtype, while only rs2647046 showed an association in multiple myeloma (P<0.1).

As a further demonstration of locus heterogeneity at 6p21.32 for FL, rs9268853, rs2647046 and rs2621416 are not in LD with any previously reported FL SNPs in this region (Table S3). A step-wise conditional logistic regression analysis of the FL SNPs from the phase-1 was performed. These investigations in Table 3 suggest some of the SNPs at the HLA locus are independent of the most significant SNP rs4530903. Hence, three SNPs rs4530903, rs9268853 and rs2621416 appear to be independent SNPs. The minor allele frequencies for these SNPs were comparable to the Hapmap population across the three stages.

**Table 3:** Conditional logistic regression analysis for the 6p21.32 SNPs in FL.

CHR	SNP	rs4530903	rs4530903-rs9268853	rs4530903-rs9268853-rs2621416
6	rs9268853	1.93×10^−8^	-	-
6	rs2647046	1.68×10^−5^	0.063	0.41
6	rs2621416	2.51×10^−4^	0.007	0.24
6	rs7453920	0.046	0.10	0.47
6	rs9276490	0.058	0.15	0.53
6	rs4530903	-	-	-

#### eQTL analysis

Analysis of the available data on expression quantitative trait loci (eQTL) on lymphoblastoid cell lines [12] for the lead SNPs and corresponding candidate genes from the GWAS was performed. In the cis- eQTLs-gene analysis, we identified eQTL in the two genes of interest based on the SNP P-values obtained from the association study using the database utility GENEVAR [13]. rs3129763 in *HLA-DQA2* showed the strongest SNP-gene association (Figure S2, Figure S3) (P = 1.23×10^−13^), while rs241440 (Figure S2, Figure S4) showed association (P = 3.3×10^−7^) with *TAP2* (Figure S2). Presence of any SNP that was in linkage disequilibrium with the expression probe was checked in HapMap data. There was a SNP rs9276442 in the expression array probe for *HLA-DQA2*. However, it is 124 kb upstream from the most significant eQTL SNP, rs3129763 and examination of haplotype blocks in the HapMap suggests that these two SNPs are not in LD. It is unlikely that the eQTL association is a consequence of perturbed probe binding due to the SNP. A lymphoma risk SNP, rs948562 identified at chromosome 11q12.1, was associated with expression levels of *OR9Q* in lymphocytes (P_adj_ = 2.49×10^−2^, adjusted using non-parametric permutation, Figure S2).

#### Shared variants

In a global analyses of all SNPs with associations at P<1×10^−3^, we observed that most SNPs were exclusive to the subtypes FL and DLBCL, thus reinforcing the notion of subtype-specific etiologic pathways. We found an overlap of only two SNPs between DLBCL and FL, confirming distinct genetic susceptibility in these subtypes. Predictably, about a half of the variants (P<10^−3^) were shared between LYM and NHL (Figure S5A). The same trend was noted in a gene-set enrichment analysis. Within the top 100 genes enriched in LYM, NHL, FL and DLBCL, we found the majority of genes associated with specific lymphoma subtypes, with a few genes common between them. One gene, *RELN*, was common to all subsets and groupings (Figure S5B). Since the sample sizes for the subtypes were small, this analysis was not done for each individual subtypes of NHL. In FL, significantly associated genes were *HLA-DOB*, *HLA-DQA2*, *TAP2*, *HLA-DRA*, *HLA-DQB1* and *HLA-DRB1* (P_FL_<4×10^−8^) and in LYM, these were *HES6*, *ILKAP*, *PER2*, *FOXP1*, *OR5* family members, and *ATF6* (P_LYM_<1×10^−5^).

## Discussion

The major finding of this study is the observation that some regions are most strongly associated with a particular subtype of lymphoma, e.g. 6p21.32 in FL, while others are most strongly associated with combined types of lymphoma, e.g. the novel regions on 11q12.1. Evidence favoring a model of common susceptibility loci includes observations of familial clustering of multiple subtypes of lymphoma. Several studies have now discovered pre-disposing genetic loci at the HLA region for FL, DLBCL, CLL and HD [4], [5], [6], [7], [8] and some of these reports highlight the existence of shared susceptibility loci at the individual subtype levels that were studied. Etiologically, patients with HD have a higher risk of developing NHL as a secondary malignancy [14]. Similarly, patients with NHL have a higher risk of developing HD at a later stage [15]. At a molecular level, the model of common susceptibility pathways is supported by recent studies examining the coding sequences and genomes of non-Hodgkin's lymphomas, which have demonstrated increased mutation burden in shared genes [16], [17]. In addition, recent tumor analysis has demonstrated that DLBCL and FL share somatic mutations in the same chromatin and histone modifying genes, *MLL2* and *MEF2B*, respectively [16]. Such evidence notwithstanding, a direct test of subtype-specific association would require a very large number of cases per subtype, feasible as part of a combined consortium approach. However, as a first approximation of shared versus subtype specific susceptibilities to lymphoma, it is possible to determine if a putative locus shows heterogeneity. For the 11q12.1 region shown here to be a pan-lymphoma susceptibility locus, there was no evidence of such heterogeneity within the largest subtypes.

Of the susceptibility markers reported here, the 6p21.32 HLA II region has been previously associated with FL and NHL [4], [5], [6]. In our report, the 6p21.32 region was implicated by three SNPS; rs4530903 upstream from *HLA-DRB1* and *HLA-DQA1*, rs2621416 upstream of *HLA-DQB2*, and rs9268853 downstream of HLA-DRA, HLA-DRB5 and *HLA-DRB1*, but upstream of *BTLN2*. rs2621416 and rs9268853 have also been associated with risk for ulcerative colitis [18] and rheumatoid arthritis [19] respectively, both of which increases risk for certain types of lymphoma. Allelic heterogeneity at this same locus has also been demonstrated in FL, with both protective and risk alleles described [6]. rs2647012, a previously reported SNP [6] is correlated (r^2^ = 1, D′ = 1) with rs2647046 in our results. None of the 6p21.32 SNPs are correlated with rs10484561, the HLA-associated SNP previously described [4]. Our data support the earlier findings of allelic heterogeneity at this region, with a slightly stronger magnitude of the effect size.

The novel regions reported here include 6p23 and 11q12.1, represented by SNPs mapping near genes with biologically plausible ties to lymphoid development. The novel SNP at 6p23, rs707824, is upstream of *JARID2*, encoding Jumonji, which co-localizes with the polycomb repressive complex 2 and *H3K27me3* on chromatin and plays a role in self-renewal and differentiation of embryonic stem cells [20]. *JARID2* is regulated by *miR-155* where very high levels decrease endogenous *JARID2* mRNA levels [21]. High levels of *miR-155* are observed in different types of B-cell lymphomas (DLBCL, HD and latency type III EBV-positive Burkitt lymphoma), and transgenic mice expressing *miR155* at the late pro-B-cell stage of differentiation developed B-cell tumors. *JARID2*/Jumonji-deficient mice have widespread developmental defects including abnormalities of hematopoiesis [22]. rs707824 is located downstream of *CD83*. CD83 antigen, also known as B-cell activation protein, is expressed on dendritic cells and is thought to have roles in the modulation of antigen presentation and CD4^+^ T cell generation [23].

The 11q12.1 region reported here was marked by two SNPs, rs948562, located within the non-coding gene *ZFP91*, and rs12289961. rs12289961 at 11q12.1 is 230 kb upstream of the *LPXN* (leupaxin) locus, originally identified binding to alpha4 integrins and playing a role in integrin-mediated cell adhesion [24]. *LPXN* was found to be a member of a fusion protein with *RUNX1* in human acute leukemia where wild-type *LPXN* was shown to transform NIH 3T3 cells [25]. Particularly relevant to its putative role suggested here in B-cell lymphomagenesis, *LPXN* is preferentially expressed in hematopoietic cells and plays an inhibitory role in B-cell antigen receptor signaling and B-cell function [26].

eQTL analysis showed that there was overlap between the most significant SNPs in the GWAS and lymphoblastoid cell lines cis-eQTL candidate genes, such as *HLA-DQA2* and *TAP2*. *HLA-DQA2* plays a pivotal role in the immune system by presenting peptides derived from extracellular proteins. Gene set enrichment analysis showed interesting candidates related to lymphomagenesis and hematopoietic cell development in the top 20 significant genes. The one variant common in all gene enrichment analyses was *RELN*, which has been shown to be recurrently mutated in acute lymphocytic leukemia [27].

Based on patterns of inheritance of multiple subtypes of lymphoid neoplasms in families, as well as from the GWAS data reported here, there is evidence to suggest that multiple phenotypes of lymphoma may be associated with shared common genetic predispositions. The candidate genes uncovered in this GWAS suggest that in addition to the genes involved in immune regulation, such as *HLA* and *JARID2*, those involved in B-cell development (e.g. *LPXN*) are logical targets for further studies. It is possible that the GWAS associations with multiple phenotypes reported here have resulted from the ascertainment utilized, since the study was enriched with a familial subset of samples. However, we included only one individual from each kindred, precluding a spurious association of a single SNP with multiple phenotypes in the same family. SNPs that show shared susceptibility, including some of those discovered here, may yet have strongest association with specific lymphoma subtypes. While this study reports associations within combined smaller subtypes, e.g. mantle cell and marginal zone lymphoma, larger sample sizes will be required to delineate whether these and other associations are shared or subtype specific.

Thus, we have described two novel lymphoma-susceptibility regions, one at 11q12.1 and another putative susceptibility locus at 6p23, and further characterized the 6p21.32 (HLA class II) association signal observed in a prior GWAS of FL. While genetic susceptibility to lymphoma has been viewed as subtype specific, here we propose an alternate model. Based on our analysis of the overlap between genotypes and phenotypes (Figure S5), we predict that the shared loci associated with multiple subtypes of lymphoma will be less frequent than subtype-specific susceptibilities. Finally, the effect sizes observed in this report (0.59–1.93) are somewhat higher than those previously reported, e.g. for breast and colon cancer, but well below thresholds required for clinical utility [28]. As in other cancer genome-wide association studies, the novel loci reported here harbor interesting genes in pathways that regulate hematopoiesis, offering potential new insights into the pathogenesis of lymphoid neoplasms.

## Methods

### Ethics statement

All cases were ascertained through Memorial Sloan-Kettering Cancer Center IRB-approved protocols, or a protocol approved by the IRB at the Dana Farber Cancer Institute or Hadassah Hebrew University (Table S4). These protocols either required informed consent for identified use of specimens for research into the genetic basis of lymphoma, or allowed research use of specimens permanently de-identified prior to genotyping.

### Sample selection for stage-1 and stage-2

The stage-1 of our study was comprised of 944 unrelated probands. This ascertainment was enriched to included 282 cases of familial lymphoproliferative syndrome, defined as two or more lymphoid cancers in the same lineage. These kindreds were characterized by mixed phenotypes of lymphoid malignancy (Figure S6), and kindreds contained from 2 to 5 affected relatives. In addition, stage-1 contained 107 cases of lymphoma with a first degree relative affected by a lymphoid malignancy, and 347 cases of early onset (age of diagnosis <45 years) lymphoma. Stage 2 was comprised of 1245 unrelated lymphoma probands from a prevalent ascertainment at MSKCC and unselected for specific histology or family history of lymphoma. Lymphomas were categorized according to a modification of the 2008 World Health Organization classification system; primary reports were obtained in all cases and reviewed by two of the authors (KO and AZ). Because of the presence of multiple subtypes in kindreds with familial lymphoma, all subtypes of B and T cell lymphoma, including Hodgkin's disease and plasma cell neoplasms were included in both stage 1 and 2, although it was recognized that sizes of these subgroups would be too small to allow subset analysis. The sample distribution of histologic subsets of lymphoma mirrors the prevalence of the disease subtypes in the US population.

### Genotyping for Stage-1 and quality control of data

Genotyping of the cases was performed utilizing the Affymetrix 6.0 SNP array. For control data, Bipolar and GENEVA Diabetes Study (NHS/HPFS) data were downloaded from dbGAP (accession phs000017.v3 http://1.usa.gov/xrXL1D and phs000091.v2 http://1.usa.gov/yevUOY). Affymetrix SNP 6.0 CEL files were arranged according to the batches in which data were originally genotyped. Data were initially quality checked for the gender and Mini-DM thresholds. Only CEL files that passed a Mini-DM >85% were used in the full Birdseed [29] genotyping of the 906,000 SNPs. The mean heterozygosity of each sample was computed (26.8) and samples with low or high heterozygosity were excluded. Samples that passed >95% Birdseed calls were further processed to generate PLINK [30] formatted files, using only calls that had copy number state two and a confidence score >0.9. This was performed using the utility Birdsuite to PLINK from Broad Institute. Hapmap controls were removed. In addition, any sample that showed abnormal copy number profile states in Birdsuite were excluded (CN0%, CN1%, CN2%, CN3% and CN4%). Particular attention was paid to any samples that had the CLL/SLL phenotype in the copy number variability screen, to exclude samples with somatic mosaicism caused by circulating tumor cells. Individuals from dbGaP marked as controls in the data-manifest were retained for further study. Samples with genetic or cryptic relatedness were excluded by using the relationship score-matrix (PI_HAT<0.1) in the entire dataset. Data was filtered for multi-mapping, mitochondrial and monomorphic SNPs on the Affymetrix 6.0 SNP Chip. Individuals and SNPs were filtered for 95% genotyping rate and departures from Hardy-Weinberg equilibrium [31]. SNPs were also removed if they failed differential missing or haplotype-based differential missing tests as implemented in PLINK. Finally, the data was matched against previously called genotyping data from dbGAP for a subset of SNPs and their allele frequencies. Analyses were carried out on 944 cases and 4044 controls on 530,583 SNPs. Principal component analysis was carried out to test for population match in both cases and controls (Figure S7). Association was performed using case-control status with each phenotype specifically defined, along with age and the first four eigenvectors from the output of EIGENSTRAT [32] program using logistic regression.

### Control data for stage-2: New York Cancer Project

Controls for the replication were gathered from the New York Cancer Project (NYCP), which is a study of 18,000 New York City residents that allows researchers to better understand how factors such as environment, lifestyle, diet, family health history, and genetics affect the development of cancer and an array of other life threatening diseases. The data include age, gender, history of cancers (including lymphoma) and ethnicity [33]. All subjects consented to use of samples to study the genetics of any disease state. Only samples with self-declared European ancestry were used for stage-2. Since individuals of Ashkenazi Jewish ethnicity formed a subset of both ascertainments, ethnicity was used as one of the covariates in the analysis in stage-2. Genotyping for stage-2 was carried out by designing multiplexed PCR using Sequenom iPLEX assays and analyzed using MassARRAY [34]. Genotypes were called using TYPER 4.0.2 software.

### Imputing SNPs at Chr6 and Chr11 loci

The dataset (BED, BIM, FAM) was split to each chromosome, then subset using gtool [35] to create .gen and .sample files. Imputation was done using pre-phasing and best-guess imputing using IMPUTE2 [36] with the references used being 1000 genomes and Hapmap3 populations for genome build v36. Best practices for imputation of the data were followed The dataset (BED, BIM, FAM) was split to each chromosome, then subset using gtool [35] to create .gen and .sample files. Imputation was done using pre-phasing and best-guess imputing using IMPUTE2 [36] with the references used being 1000 genomes and Hapmap3 populations for genome build v36. Best practices for imputation of the data were followed [37]. The dosage output was filtered for confidence scores and analyzed using PLINK, filtered on INFO and plotted using locuszoom [38]. Haplotypes were viewed in Haploview [39]. The dosage output was filtered for confidence scores and analyzed using PLINK, filtered on INFO and plotted using locuszoom [38]. Haplotypes were viewed in Haploview [39].

### Selection of SNPs into Stage-2

SNPs were ranked on p-value in both major types and subtype specific analyses. Each index-ranked SNP (within top 100 SNPs) was graded based on a custom script used to generate scatterplots from Birdsuite, which were inspected and graded on the cluster separation and skew. In order to prioritize the SNPs that were to be replicated, SNPs were given a negative grade if they were singletons (i.e. neighboring SNPs not showing low p-values). A positive grade was given if a given SNP showed low p-value (P<5×10^−4^) in any other type or subtype. Only SNPs with good scatterplots were selected for the iPLEX design. Analysis was performed by logistic regression using the same criteria as stage-1, however, instead of the PCA, self-reported ethnicity information was used. Only Caucasian samples were used in the replication study. A meta-analysis of the stage-1 and stage-2 data was performed using the results of the logistic regression. For test of heterogeneity specifically for the 6p21.32 locus, the combined dataset consisting of stage-1 and stage-2 was split into three major groups namely FL, DLBCL and any other NHL subgroup designated as NFD in this report. Since we have only one control set, the control samples were randomly assigned in a fixed ratio to match the percent cases per subset without replacement. The three clusters were joined together to perform Breslow-Day test using PLINK.

### Gene set enrichment analysis

We performed gene set enrichment analysis using the p-values from each of the subgroup and group analyses. The program VEGAS [40] was used to compute the gene enrichment analyses. It annotates SNPs to corresponding genes (±50 kb boundaries), produces a gene-based test statistic, and then uses simulation to calculate an empirical gene-based p-value. The Hapmap population was used as a reference. The top 10 percent of significant SNPs were chosen for the analysis with simulation performed 10^6^ times. Venn diagram was created using Venny (http://bioinfogp.cnb.csic.es/tools/venny/index.html).

### eQTL analysis in lymphoblastoid cell line

We analyzed available hapmap3 population data from lymphoblastoid cell lines [12] for eQTLs [12] using GENEVAR [13]. Two types of analyses were performed, (1) identifying cis-eQTLs in candidate genes discovered from the GWAS and (2) SNP-gene association analysis. Adjusted p-values (P_adj_) were derived from 10,000 permutations as implemented on the GENEVAR applet.

## Supporting Information

Figure S1 Power calculations for the GWAS stage-1. Calculations were performed assuming effective degrees of freedom of 500,000, and (Panel A) 944 LYM cases with a control to case ratio of 4 and LD value between 0.7–0.8; (Panel B) 275 FL cases with a control to case ratio of 15 and LD value between 0.7–0.8. Power was varied between 70 and 80%. As observed, for LYM, the detectable relative risk (RR1) is stable around 1.5 or greater (Panel A) and for FL, the RR1 varies (1.8–2.2) for the marker allele frequencies studied.
(TIF)

Figure S2 eQTL from lymphoblastoid cell lines for SNPs and candidate genes in our GWAS. (Panel A) rs3129763 in HLA-DQA2 showed the best SNP-gene association (P = 1.23×10^−13^), while rs241440 shows (Panel B) association (P = 3.3×10^−7^) with TAP2. (Panel C) One of the SNPs in the GWAS, rs948562 showed permutation p-value (P_adj_) = 2.49×10^−2^ in lymphocytes for the gene OR9Q2 in the Chr11q12 locus.
(TIF)

Figure S3 Boxplots of all SNPs associated with candidate gene HLA-DQA2.
(TIF)

Figure S4 Boxplots of all SNPs associated with candidate gene OR9Q2.
(TIF)

Figure S5 Overlap and distinct SNPs (A) and genes (B) amongst each category of LYM, NHL, FL, DLBCL. Top 100 genes from each gene-enrichment analysis and the SNPs with p<1.0×10^−3^ were used for comparison.
(TIF)

Figure S6 Pedigrees of two representative families with familial lymphoproliferative syndrome included in our stage-1. (LPS, defined as two or more lymphoid cancers in the same parental lineage), showing occurrence of multiple subtypes within the same individuals and the sibships. Lym-NOS = Lymphoma, not otherwise specified. Leuk = Leukemia.
(TIF)

Figure S7 Principal component analysis (PCA) plot shows the overlap of cases and controls. The groups included are GAIN Bipolar controls (BPC), GENEVA Diabetes controls (DBT) and lymphoma cases (LYM). This PCA plot shows the Caucasian and Jewish clusters distinguished using the first two major PCs. PCA was done on a subset of LD pruned SNPs from the original dataset. The top four eigenvectors were used for adjusting population stratification in the association analysis. Analysis was done using EIGENSTRAT.
(TIF)

Table S1 SNPs selected from the GWAS to perform replication.
(DOCX)

Table S2 Results of the replication phase (Stage-2).
(DOCX)

Table S3 Correlation and recombination rate of SNPs associated with the HLA and 11q12.1 regions in HapMap.
(DOCX)

Table S4 Sample sizes in Stage-1 and Stage-2. The samples for the phase 1 were collected at MSKCC (N = 860), Dana Farber Cancer Institute (N = 74) and Hadassah Hebrew University, Israel (N = 10). The replication phase was ascertained at MSKCC.
(DOCX)
