# Supplementary figures and images for: Susceptibility Loci Associated with Specific and Shared Subtypes of Lymphoid Malignancies
Source: PLoS Genet. 2013 Jan 17;9(1):e1003220. doi: 10.1371/journal.pgen.1003220 (PMC3547842; doi:10.1371/journal.pgen.1003220)

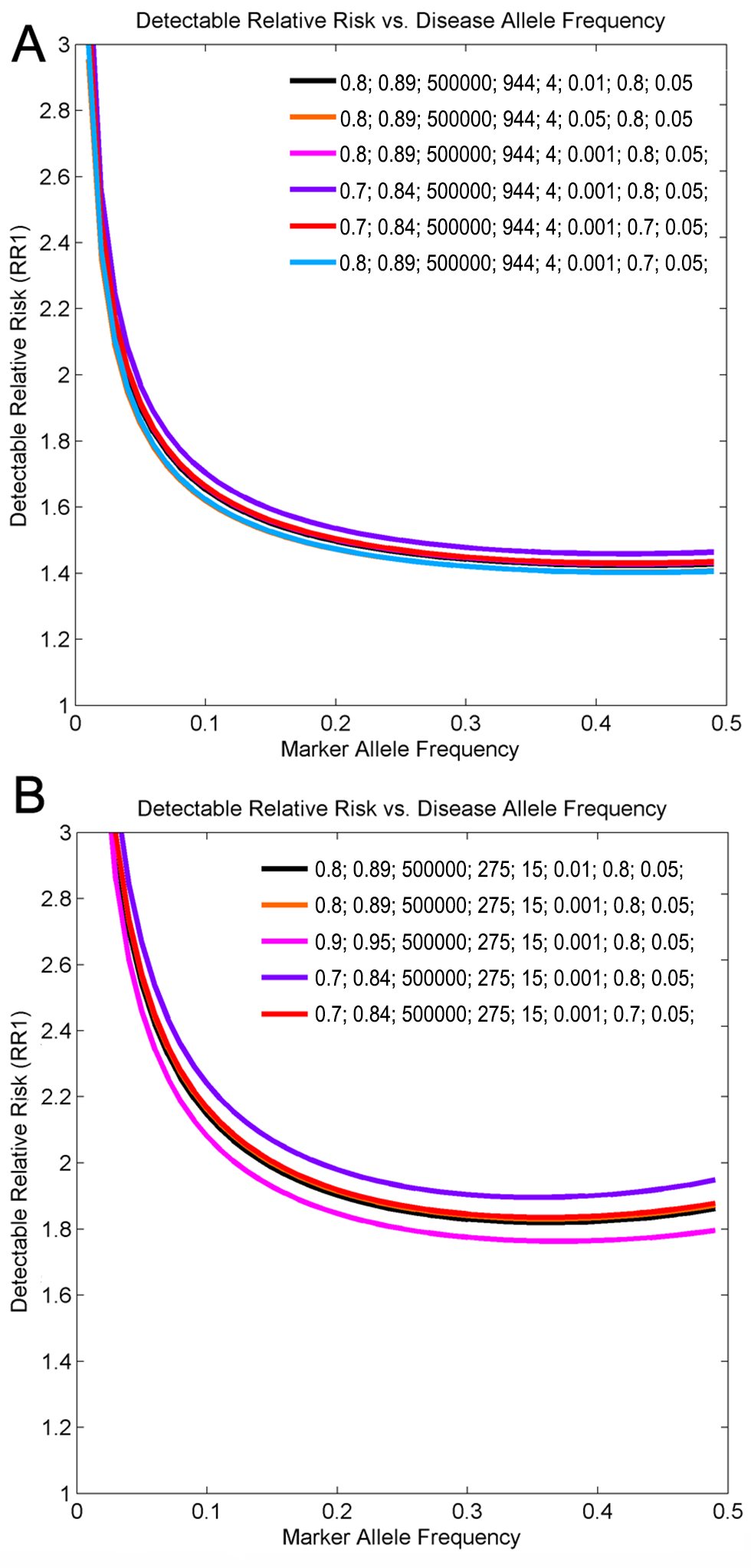

Supplement: Figure S1 — Power calculations for the GWAS stage-1. Calculations were performed assuming effective degrees of freedom of 500,000, and (Panel A) 944 LYM cases with a control to case ratio of 4 and LD value between 0.7–0.8; (Panel B) 275 FL cases with a control to case ratio of 15 and LD value between 0.7–0.8. Power was varied between 70 and 80%. As observed, for LYM, the detectable relative risk (RR1) is stable around 1.5 or greater (Panel A) and for FL, the RR1 varies (1.8–2.2) for the marker allele frequencies studied. (TIF) [file pgen.1003220.s001.tif]

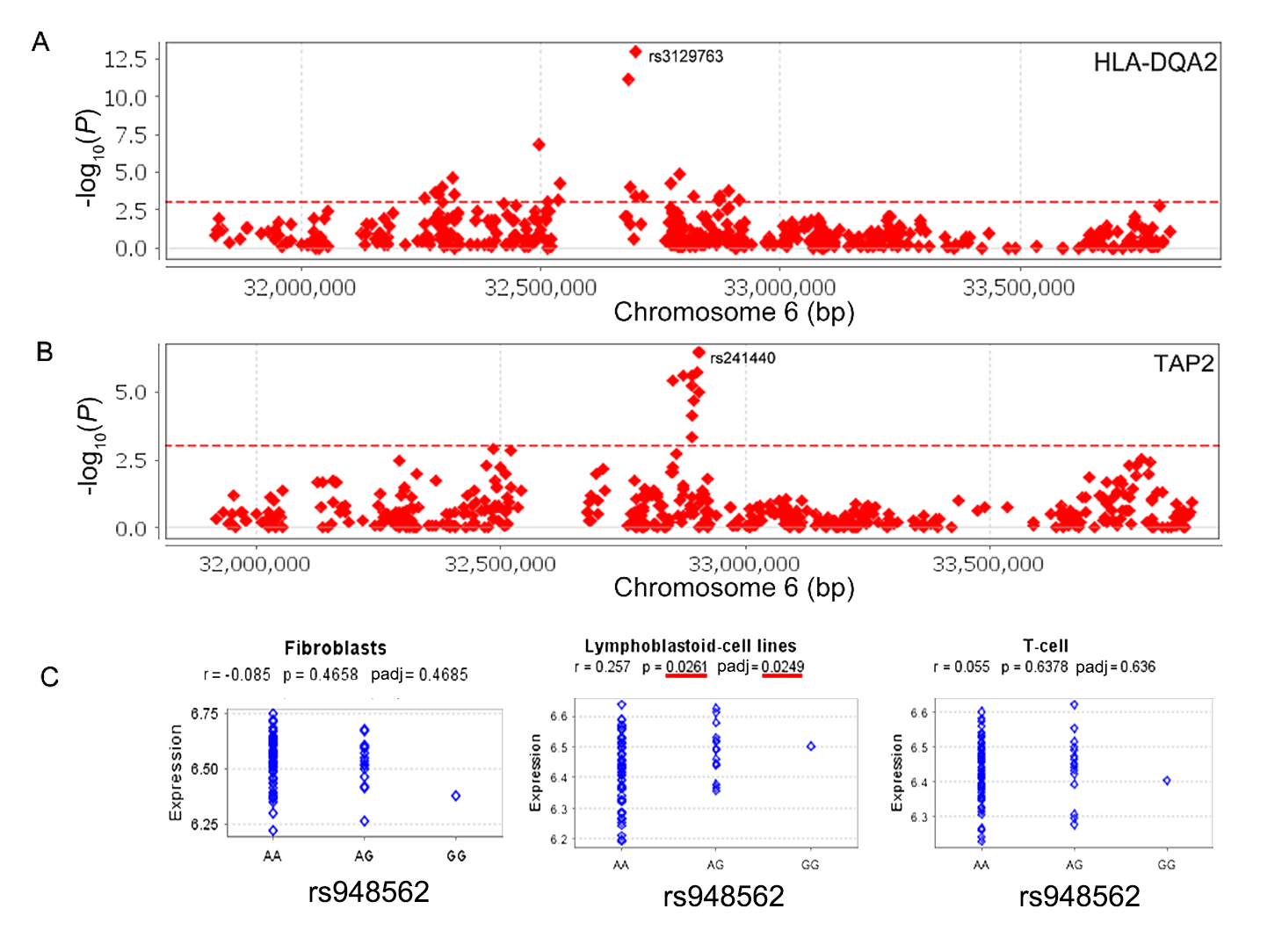

Supplement: Figure S2 — eQTL from lymphoblastoid cell lines for SNPs and candidate genes in our GWAS. (Panel A) rs3129763 in HLA-DQA2 showed the best SNP-gene association (P = 1.23×10^−13^), while rs241440 shows (Panel B) association (P = 3.3×10^−7^) with TAP2. (Panel C) One of the SNPs in the GWAS, rs948562 showed permutation p-value (Padj) = 2.49×10^−2^ in lymphocytes for the gene OR9Q2 in the Chr11q12 locus. (TIF) [file pgen.1003220.s002.tif]

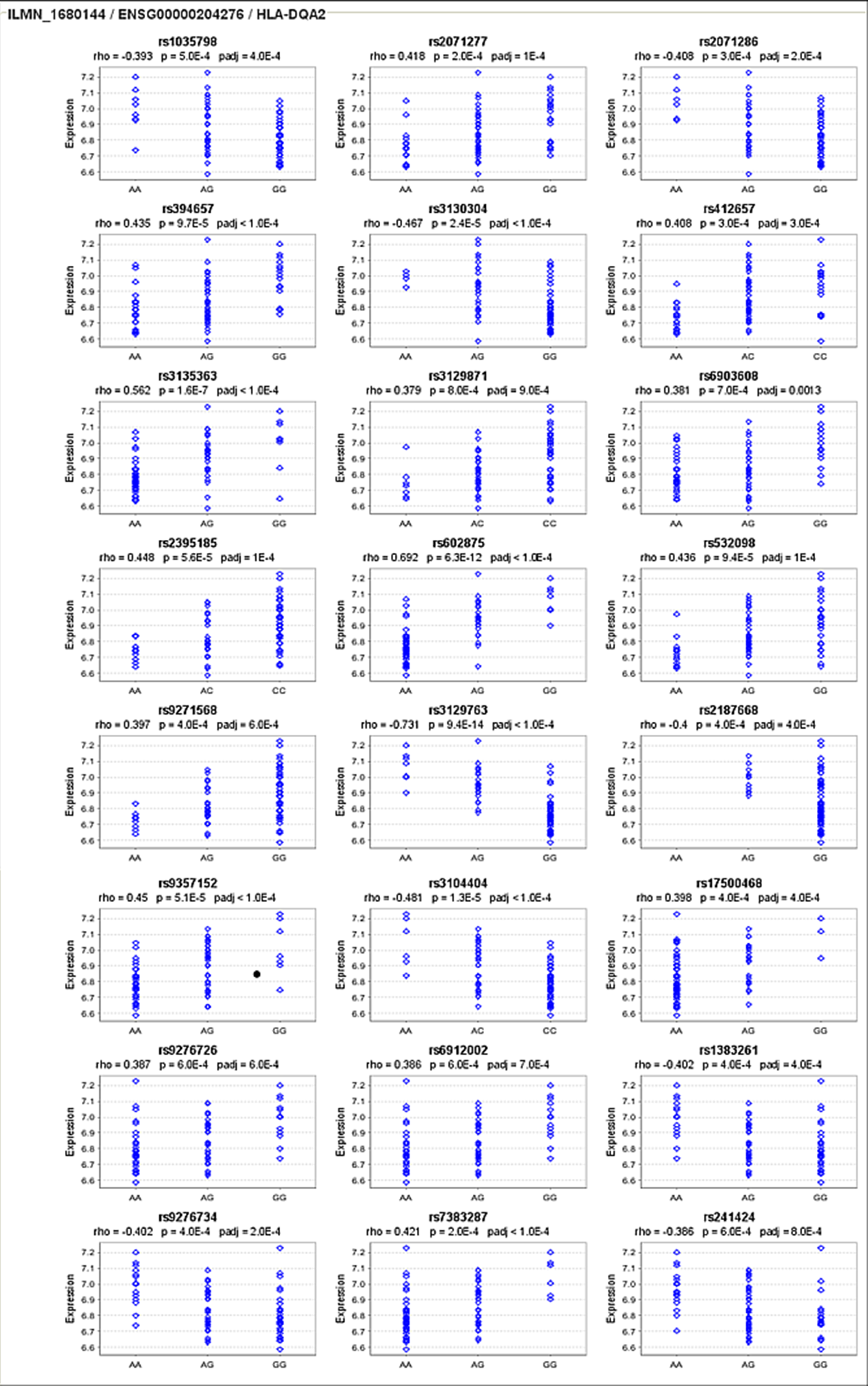

Supplement: Figure S3 — Boxplots of all SNPs associated with candidate gene HLA-DQA2. (TIF) [file pgen.1003220.s003.tif]

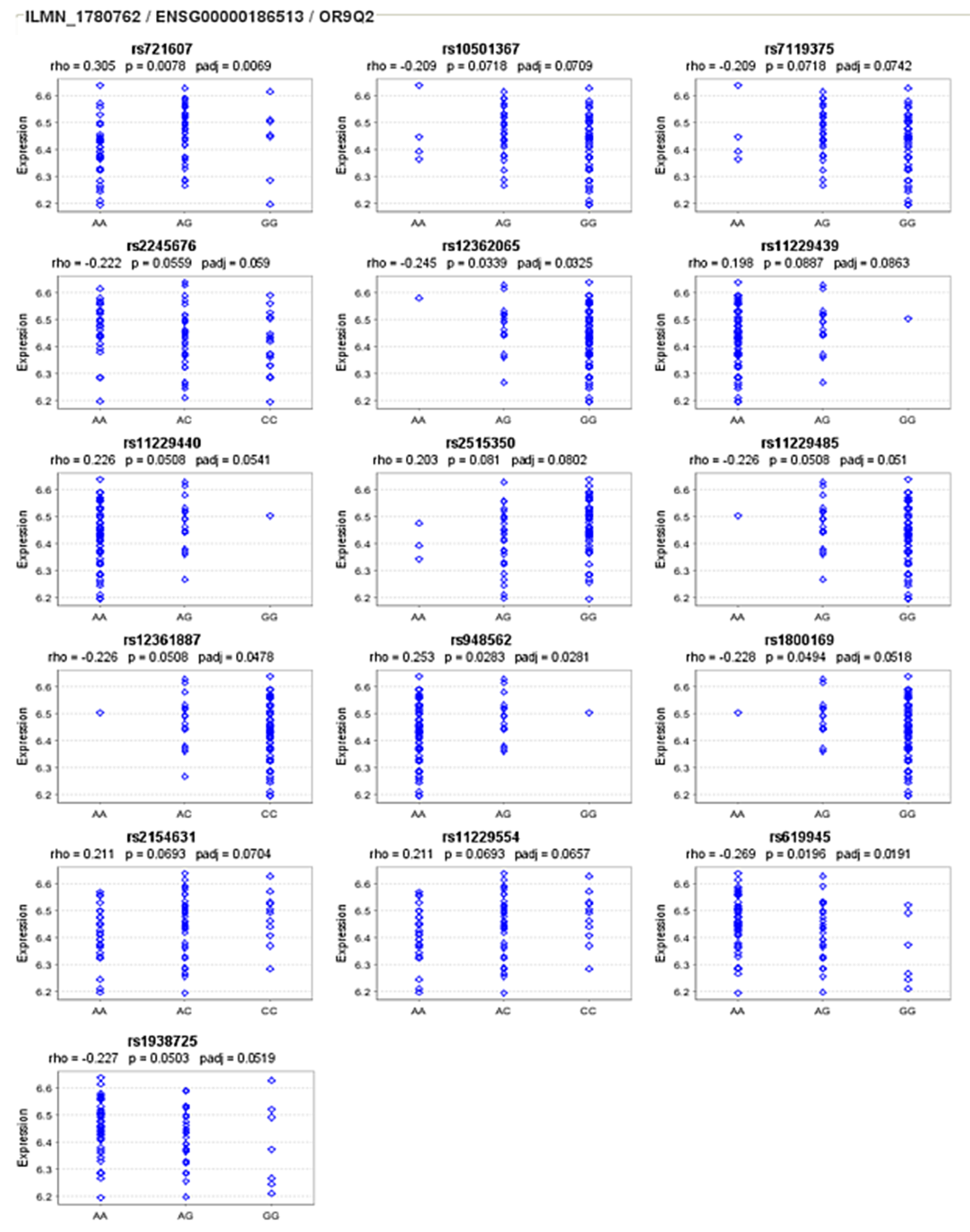

Supplement: Figure S4 — Boxplots of all SNPs associated with candidate gene OR9Q2. (TIF) [file pgen.1003220.s004.tif]

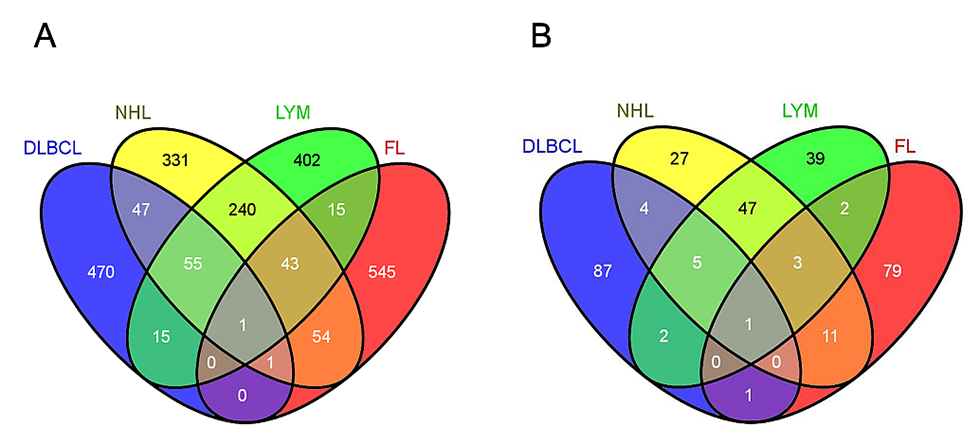

Supplement: Figure S5 — Overlap and distinct SNPs (A) and genes (B) amongst each category of LYM, NHL, FL, DLBCL. Top 100 genes from each gene-enrichment analysis and the SNPs with p<1.0×10^−3^ were used for comparison. (TIF) [file pgen.1003220.s005.tif]

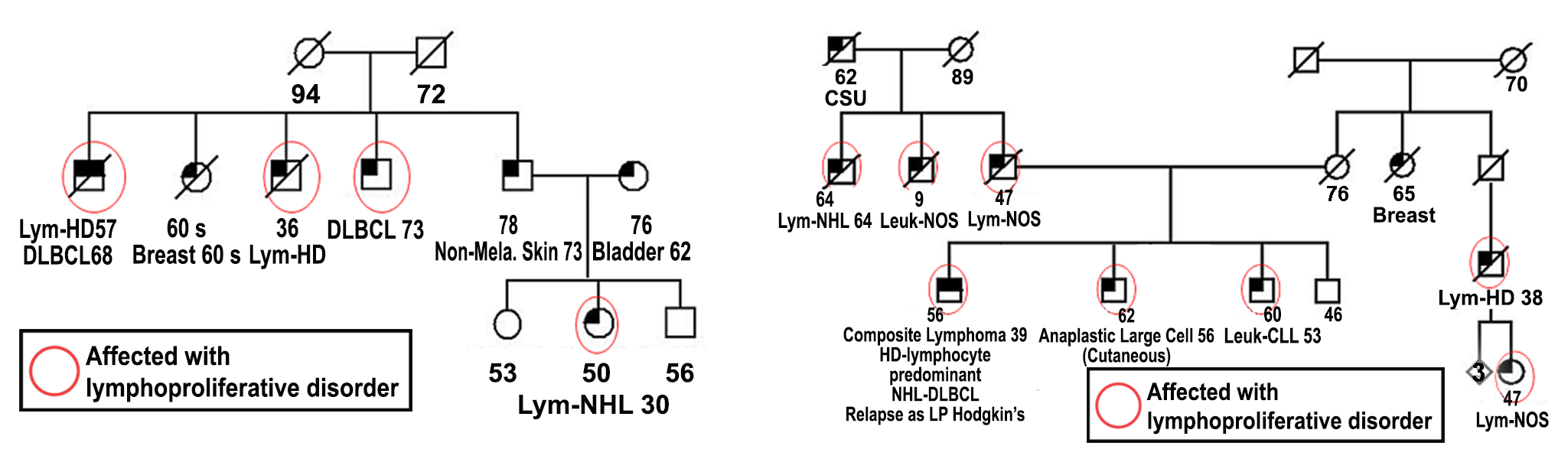

Supplement: Figure S6 — Pedigrees of two representative families with familial lymphoproliferative syndrome included in our stage-1. (LPS, defined as two or more lymphoid cancers in the same parental lineage), showing occurrence of multiple subtypes within the same individuals and the sibships. Lym-NOS = Lymphoma, not otherwise specified. Leuk = Leukemia. (TIF) [file pgen.1003220.s006.tif]

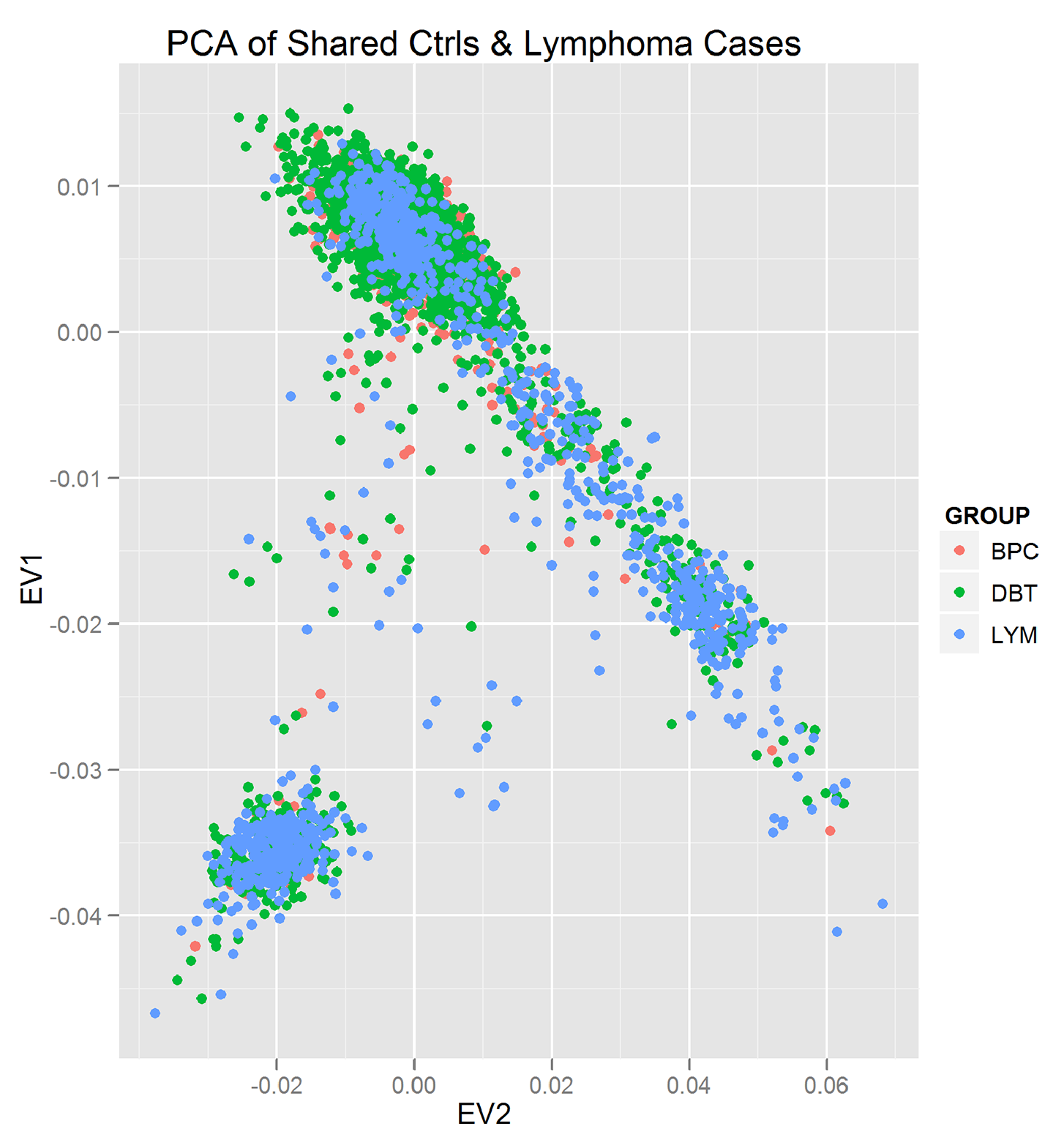

Supplement: Figure S7 — Principal component analysis (PCA) plot shows the overlap of cases and controls. The groups included are GAIN Bipolar controls (BPC), GENEVA Diabetes controls (DBT) and lymphoma cases (LYM). This PCA plot shows the Caucasian and Jewish clusters distinguished using the first two major PCs. PCA was done on a subset of LD pruned SNPs from the original dataset. The top four eigenvectors were used for adjusting population stratification in the association analysis. Analysis was done using EIGENSTRAT. (TIF) [file pgen.1003220.s007.tif]
