# Supplementary material for: Susceptibility Loci Associated with Specific and Shared Subtypes of Lymphoid Malignancies
Source: PLoS Genet. 2013 Jan 17;9(1):e1003220. doi: 10.1371/journal.pgen.1003220 (PMC3547842; doi:10.1371/journal.pgen.1003220)
Supplement: Table S1 — SNPs selected from the GWAS to perform replication. (DOCX) [file pgen.1003220.s008.docx]

**Table S1:** SNPs selected from the GWAS to perform Replication

| **Replication Phase** | | | **Lym** | | **Nhl** | | **FL** | | **Dlbcl** | | **NFD** | |
| --- | --- | --- | --- | --- | --- | --- | --- | --- | --- | --- | --- | --- |
| **Chr** | **SNP** | **RA** | **OR (CI)** | **P** | **OR (CI)** | **P** | **OR (CI)** | **P** | **OR (CI)** | **P** | **OR (CI)** | **P** |
| 1 | rs11581364 | T | 1.29 (1.15 - 1.45) | 2.002E-05 | 1.29 (1.13 - 1.46) | 0.0001655 | 1.21 (1 - 1.46) | 0.05537 | 1.34 (1.1 - 1.62) | 0.003729 | 1.34 (1.14 - 1.57) | 0.0004145 |
| 1 | rs2762682 | T | 0.74 (0.65 - 0.85) | 0.0000113 | 0.74 (0.64 - 0.86) | 5.652E-05 | 0.87 (0.7 - 1.08) | 0.1938 | 0.6 (0.47 - 0.76) | 1.622E-05 | 0.75 (0.63 - 0.9) | 0.002362 |
| 1 | rs2985340 | C | 0.75 (0.66 - 0.86) | 3.158E-05 | 0.76 (0.65 - 0.88) | 0.0002206 | 0.9 (0.72 - 1.11) | 0.3087 | 0.61 (0.48 - 0.77) | 2.639E-05 | 0.76 (0.63 - 0.91) | 0.003345 |
| 1 | rs3767635 | G | 1.33 (1.18 - 1.5) | 3.792E-06 | 1.29 (1.12 - 1.47) | 0.0002529 | 1.12 (0.92 - 1.36) | 0.2722 | 1.35 (1.11 - 1.65) | 0.003129 | 1.46 (1.24 - 1.72) | 5.234E-06 |
| 2 | rs13020362 | G | 1.36 (1.2 - 1.54) | 1.608E-06 | 1.33 (1.16 - 1.52) | 5.922E-05 | 1.36 (1.11 - 1.66) | 0.003023 | 1.35 (1.1 - 1.66) | 0.003734 | 1.28 (1.08 - 1.52) | 0.005027 |
| 2 | rs1430261 | G | 0.63 (0.51 - 0.79) | 3.533E-05 | 0.6 (0.47 - 0.77) | 5.147E-05 | 0.61 (0.43 - 0.88) | 0.008523 | 0.59 (0.4 - 0.86) | 0.005833 | 0.69 (0.52 - 0.93) | 0.01525 |
| 2 | rs840947 | A | 0.92 (0.82 - 1.03) | 0.1576 | 0.87 (0.77 - 0.99) | 0.03487 | 1.02 (0.85 - 1.23) | 0.8436 | 0.63 (0.52 - 0.77) | 6.488E-06 | 1.09 (0.93 - 1.28) | 0.2795 |
| 3 | rs4677602 | T | 1.33 (1.18 - 1.5) | 3.289E-06 | 1.38 (1.21 - 1.58) | 2.845E-06 | 1.52 (1.25 - 1.85) | 2.575E-05 | 1.29 (1.06 - 1.58) | 0.01156 | 1.21 (1.02 - 1.42) | 0.02727 |
| 3 | rs4680035 | G | 1.32 (1.18 - 1.48) | 1.558E-06 | 1.35 (1.19 - 1.54) | 3.24E-06 | 1.31 (1.09 - 1.58) | 0.003828 | 1.21 (1 - 1.46) | 0.04957 | 1.43 (1.22 - 1.68) | 8.675E-06 |
| 3 | rs4855535 | G | 1.44 (1.23 - 1.7) | 7.917E-06 | 1.46 (1.22 - 1.75) | 3.956E-05 | 1.33 (1.01 - 1.75) | 0.04425 | 1.5 (1.15 - 1.96) | 0.002912 | 1.56 (1.25 - 1.94) | 7.949E-05 |
| 3 | rs528581 | C | 0.76 (0.67 - 0.85) | 5.119E-06 | 0.74 (0.65 - 0.85) | 9.938E-06 | 0.79 (0.66 - 0.96) | 0.01611 | 0.82 (0.68 - 1) | 0.04821 | 0.69 (0.59 - 0.82) | 1.292E-05 |
| 3 | rs7644066 | G | 1.34 (1.19 - 1.51) | 1.914E-06 | 1.38 (1.21 - 1.58) | 2.012E-06 | 1.54 (1.26 - 1.87) | 1.713E-05 | 1.31 (1.08 - 1.6) | 0.007569 | 1.21 (1.02 - 1.43) | 0.02508 |
| 4 | rs2712136 | G | 0.78 (0.7 - 0.88) | 4.063E-05 | 0.75 (0.66 - 0.85) | 1.348E-05 | 0.73 (0.6 - 0.88) | 0.001093 | 0.73 (0.6 - 0.89) | 0.002013 | 0.85 (0.73 - 1) | 0.05407 |
| 5 | rs1115728 | C | 1.13 (1 - 1.27) | 0.04788 | 1.19 (1.05 - 1.36) | 0.008428 | 1.59 (1.31 - 1.92) | 2.991E-06 | 1.05 (0.86 - 1.28) | 0.6247 | 0.92 (0.78 - 1.09) | 0.3344 |
| 5 | rs11744200 | C | 1.4 (1.18 - 1.66) | 9.547E-05 | 1.49 (1.23 - 1.8) | 3.996E-05 | 1.18 (0.87 - 1.59) | 0.2929 | 1.78 (1.37 - 2.31) | 1.811E-05 | 1.3 (1.02 - 1.65) | 0.03095 |
| 5 | rs16882931 | T | 0.63 (0.5 - 0.78) | 2.649E-05 | 0.6 (0.47 - 0.77) | 4.191E-05 | 0.59 (0.41 - 0.85) | 0.004905 | 0.6 (0.41 - 0.87) | 0.006711 | 0.65 (0.48 - 0.88) | 0.005442 |
| 6 | rs7453920 | A | 0.8 (0.71 - 0.91) | 0.0003957 | 0.78 (0.68 - 0.89) | 0.0003268 | 0.69 (0.56 - 0.86) | 0.0006797 | 0.75 (0.61 - 0.92) | 0.007049 | 0.91 (0.77 - 1.08) | 0.2689 |
| 6 | rs1273725 | A | 1.39 (1.17 - 1.65) | 0.0001954 | 1.46 (1.21 - 1.77) | 9.203E-05 | 1.32 (1 - 1.74) | 0.0537 | 1.84 (1.42 - 2.39) | 4.137E-06 | 1.09 (0.85 - 1.4) | 0.5073 |
| 6 | rs13199524 | T | 1.18 (0.97 - 1.44) | 0.09914 | 1.35 (1.09 - 1.67) | 0.005238 | 1.87 (1.42 - 2.47) | 9.447E-06 | 1.03 (0.73 - 1.44) | 0.88 | 0.9 (0.67 - 1.21) | 0.4784 |
| 6 | rs2621416 | G | 1.24 (1.09 - 1.41) | 0.001065 | 1.42 (1.23 - 1.64) | 1.238E-06 | 1.82 (1.48 - 2.23) | 8.689E-09 | 1.37 (1.11 - 1.69) | 0.003906 | 0.94 (0.78 - 1.13) | 0.5126 |
| 6 | rs2647046 | A | 0.83 (0.74 - 0.94) | 0.003682 | 0.73 (0.64 - 0.84) | 1.331E-05 | 0.53 (0.42 - 0.66) | 3.971E-08 | 0.87 (0.71 - 1.07) | 0.1765 | 1.04 (0.88 - 1.23) | 0.6247 |
| 6 | rs4530903 | T | 1.29 (1.09 - 1.53) | 0.003508 | 1.51 (1.26 - 1.82) | 9.416E-06 | 2.01 (1.57 - 2.57) | 3.29E-08 | 1.36 (1.02 - 1.79) | 0.03383 | 0.86 (0.66 - 1.12) | 0.2695 |
| 6 | rs6459451 | C | 0.74 (0.65 - 0.84) | 6.412E-06 | 0.74 (0.63 - 0.85) | 4.071E-05 | 0.8 (0.65 - 0.99) | 0.04348 | 0.68 (0.55 - 0.86) | 0.0009231 | 0.77 (0.64 - 0.92) | 0.004374 |
| 6 | rs707824 | T | 1.26 (1.06 - 1.5) | 0.008708 | 1.39 (1.15 - 1.68) | 0.0007981 | 1.89 (1.46 - 2.45) | 1.666E-06 | 1.29 (0.97 - 1.71) | 0.08264 | 0.93 (0.72 - 1.2) | 0.5983 |
| 6 | rs9268402 | A | 1.13 (1.01 - 1.27) | 0.03139 | 1.28 (1.12 - 1.45) | 0.0001822 | 1.67 (1.38 - 2.02) | 1.413E-07 | 1.12 (0.93 - 1.35) | 0.2354 | 0.86 (0.73 - 1.01) | 0.06281 |
| 6 | rs9268853 | C | 1.14 (1.01 - 1.28) | 0.03677 | 1.27 (1.11 - 1.45) | 0.0004497 | 1.74 (1.43 - 2.1) | 1.762E-08 | 1 (0.81 - 1.22) | 0.9838 | 0.93 (0.78 - 1.11) | 0.4103 |
| 6 | rs9275572 | A | 0.84 (0.75 - 0.95) | 0.005377 | 0.76 (0.66 - 0.87) | 7.772E-05 | 0.56 (0.45 - 0.69) | 1.271E-07 | 0.88 (0.72 - 1.07) | 0.1998 | 1.03 (0.88 - 1.22) | 0.6914 |
| 6 | rs9391858 | G | 1.15 (0.97 - 1.37) | 0.1136 | 1.39 (1.16 - 1.68) | 0.0004941 | 2.03 (1.57 - 2.62) | 8.406E-08 | 1.31 (0.99 - 1.74) | 0.05975 | 0.61 (0.45 - 0.82) | 0.001102 |
| 6 | rs9446171 | G | 1.29 (1.13 - 1.48) | 0.0002193 | 1.32 (1.13 - 1.53) | 0.0003619 | 1.15 (0.92 - 1.44) | 0.2104 | 1.63 (1.3 - 2.02) | 1.479E-05 | 1.2 (1 - 1.44) | 0.0511 |
| 6 | rs9276490 | A | 0.8 (0.71 - 0.91) | 0.0004289 | 0.78 (0.68 - 0.89) | 0.0002649 | 0.7 (0.57 - 0.86) | 0.000833 | 0.74 (0.6 - 0.91) | 0.004309 | 0.91 (0.77 - 1.08) | 0.2947 |
| 7 | rs13233227 | C | 0.78 (0.69 - 0.89) | 0.0001014 | 0.75 (0.65 - 0.86) | 3.481E-05 | 0.7 (0.57 - 0.86) | 0.0005609 | 0.79 (0.65 - 0.97) | 0.02354 | 0.83 (0.7 - 0.98) | 0.02831 |
| 7 | rs1501409 | T | 1.29 (1.12 - 1.49) | 0.0003351 | 1.35 (1.15 - 1.57) | 0.0001788 | 1.74 (1.4 - 2.17) | 5.291E-07 | 1.27 (1 - 1.6) | 0.04851 | 1.02 (0.84 - 1.25) | 0.8462 |
| 7 | rs281880 | A | 1.53 (1.27 - 1.86) | 1.288E-05 | 1.57 (1.27 - 1.95) | 3.373E-05 | 1.36 (0.99 - 1.87) | 0.05719 | 1.6 (1.18 - 2.18) | 0.002702 | 1.64 (1.27 - 2.12) | 0.0001298 |
| 8 | rs1512384 | T | 1.37 (1.08 - 1.72) | 0.008498 | 1.48 (1.15 - 1.9) | 0.002561 | 1.01 (0.67 - 1.53) | 0.9477 | 2.39 (1.72 - 3.31) | 1.683E-07 | 1.1 (0.78 - 1.54) | 0.5958 |
| 9 | rs700983 | T | 1.31 (1.15 - 1.5) | 5.017E-05 | 1.39 (1.2 - 1.61) | 0.0000126 | 1.24 (0.99 - 1.54) | 0.0603 | 1.3 (1.05 - 1.62) | 0.01805 | 1.39 (1.16 - 1.66) | 0.0003293 |
| 9 | rs7026635 | G | 0.73 (0.63 - 0.84) | 1.608E-05 | 0.75 (0.64 - 0.88) | 0.0003709 | 0.7 (0.55 - 0.89) | 0.003725 | 0.72 (0.57 - 0.92) | 0.007415 | 0.73 (0.6 - 0.89) | 0.002006 |
| 10 | rs11254308 | T | 1.16 (1.03 - 1.3) | 0.01293 | 1.24 (1.09 - 1.41) | 0.001214 | 1.57 (1.29 - 1.9) | 5.458E-06 | 1.07 (0.88 - 1.3) | 0.4892 | 1.01 (0.86 - 1.19) | 0.9154 |
| 10 | rs12244831 | C | 1.13 (0.99 - 1.29) | 0.07443 | 1.26 (1.09 - 1.45) | 0.002102 | 1.66 (1.35 - 2.05) | 1.382E-06 | 1.02 (0.82 - 1.28) | 0.848 | 0.91 (0.75 - 1.1) | 0.3327 |
| 11 | rs11212484 | A | 0.7 (0.6 - 0.81) | 4.279E-06 | 0.66 (0.56 - 0.79) | 3.804E-06 | 0.77 (0.6 - 0.99) | 0.03882 | 0.65 (0.5 - 0.85) | 0.001373 | 0.69 (0.55 - 0.85) | 0.0006738 |
| 11 | rs12289961 | T | 1.37 (1.19 - 1.57) | 9.63E-06 | 1.42 (1.21 - 1.65) | 9.36E-06 | 1.45 (1.16 - 1.81) | 0.001153 | 1.19 (0.94 - 1.5) | 0.1462 | 1.45 (1.2 - 1.74) | 0.000105 |
| 11 | rs948562 | G | 1.35 (1.17 - 1.57) | 5.715E-05 | 1.45 (1.23 - 1.7) | 8.701E-06 | 1.41 (1.11 - 1.79) | 0.004381 | 1.37 (1.08 - 1.74) | 0.01051 | 1.34 (1.1 - 1.64) | 0.004244 |
| 12 | rs10843211 | G | 0.77 (0.68 - 0.88) | 0.0001554 | 0.72 (0.62 - 0.84) | 2.363E-05 | 0.74 (0.59 - 0.92) | 0.007164 | 0.66 (0.52 - 0.82) | 0.0003101 | 0.88 (0.74 - 1.06) | 0.1742 |
| 13 | rs9561965 | G | 0.67 (0.54 - 0.84) | 0.0004182 | 0.6 (0.47 - 0.78) | 7.267E-05 | 0.45 (0.3 - 0.67) | 8.191E-05 | 0.66 (0.46 - 0.95) | 0.02379 | 0.85 (0.64 - 1.12) | 0.2367 |
| 14 | rs10133918 | T | 1.04 (0.93 - 1.17) | 0.5013 | 1.09 (0.96 - 1.25) | 0.1888 | 1.62 (1.33 - 1.97) | 1.689E-06 | 0.82 (0.67 - 1) | 0.04805 | 0.95 (0.8 - 1.12) | 0.5029 |
| 14 | rs1241129 | G | 0.7 (0.59 - 0.84) | 6.913E-05 | 0.65 (0.53 - 0.79) | 1.319E-05 | 0.71 (0.53 - 0.94) | 0.01572 | 0.69 (0.52 - 0.92) | 0.01201 | 0.69 (0.54 - 0.88) | 0.002357 |
| 14 | rs17308862 | T | 0.8 (0.7 - 0.92) | 0.00169 | 0.78 (0.67 - 0.91) | 0.00189 | 1 (0.81 - 1.25) | 0.9822 | 0.56 (0.44 - 0.72) | 6.728E-06 | 0.8 (0.66 - 0.97) | 0.02397 |
| 14 | rs2356911 | G | 1.02 (0.91 - 1.15) | 0.7161 | 1.07 (0.94 - 1.22) | 0.3248 | 1.55 (1.28 - 1.89) | 1.221E-05 | 0.79 (0.65 - 0.97) | 0.02204 | 0.95 (0.8 - 1.11) | 0.5074 |
| 16 | rs1117412 | C | 1.23 (1.09 - 1.38) | 0.0006315 | 1.29 (1.13 - 1.47) | 0.0001149 | 1.5 (1.23 - 1.82) | 4.315E-05 | 1.32 (1.08 - 1.6) | 0.00579 | 1.05 (0.9 - 1.24) | 0.5156 |
| 17 | rs9303035 | G | 0.79 (0.69 - 0.9) | 0.0004004 | 0.77 (0.66 - 0.89) | 0.0004272 | 0.91 (0.74 - 1.13) | 0.3924 | 0.57 (0.45 - 0.73) | 5.462E-06 | 0.86 (0.72 - 1.03) | 0.09612 |
| 21 | rs235385 | C | 1.21 (1.07 - 1.36) | 0.002373 | 1.26 (1.1 - 1.44) | 0.000671 | 1.12 (0.92 - 1.37) | 0.2526 | 1.58 (1.29 - 1.93) | 9.515E-06 | 1.09 (0.92 - 1.29) | 0.3242 |

LYM=All lymphoma, NHL=Non-Hodgkin’s lymphoma, FL=follicular lymphoma and DLBCL=diffuse large B cell lymphoma, NFD=Non-follicular and non-diffuse large B cell subtypes. SNP=rsID of the single nucleotide polymorphism, Chr=chromosome, RA=risk allele, OR (CI)=odds-ratio (Confidence interval, lower-upper).
