# Supplementary material for: Susceptibility Loci Associated with Specific and Shared Subtypes of Lymphoid Malignancies
Source: PLoS Genet. 2013 Jan 17;9(1):e1003220. doi: 10.1371/journal.pgen.1003220 (PMC3547842; doi:10.1371/journal.pgen.1003220)
Supplement: Table S2 — Results of the replication phase (Stage-2). (DOCX) [file pgen.1003220.s009.docx]

**Table.S2:** Results of the replication Phase (stage-2)

| **Marker Details** | | | **LYM** | | **NHL** | | **FL** | | **DLBCL** | | **NFD** | |
| --- | --- | --- | --- | --- | --- | --- | --- | --- | --- | --- | --- | --- |
| ***Chr*** | ***SNP*** | ***RA*** | ***P*** | ***OR (CI)*** | ***P*** | ***OR (CI)*** | ***P*** | ***OR (CI)*** | ***P*** | ***OR (CI)*** | ***P*** | ***OR (CI)*** |
| 1 | rs2762682 | T | 1.75E-01 | 0.92 (0.82 - 1.04) | 2.26E-01 | 0.93 (0.83 - 1.05) | 4.31E-01 | 0.91 (0.72 - 1.15) | 3.73E-01 | 0.92 (0.77 - 1.10) | 3.00E-01 | 0.93 (0.81 - 1.07) |
| 1 | rs2985340 | C | 1.50E-01 | 0.92 (0.82 - 1.03) | 2.03E-01 | 0.93 (0.83 - 1.04) | 4.74E-01 | 0.92 (0.72 - 1.16) | 3.65E-01 | 0.92 (0.77 - 1.10) | 2.50E-01 | 0.92 (0.81 - 1.06) |
| 1 | rs3767635 | G | 7.06E-01 | 0.98 (0.88 - 1.09) | 6.06E-01 | 0.97 (0.87 - 1.08) | 6.89E-02 | 1.22 (0.98 - 1.52) | 8.09E-02 | 0.86 (0.72 - 1.02) | 6.46E-01 | 0.97 (0.86 - 1.10) |
| 1 | rs11581364 | T | 4.42E-01 | 0.96 (0.86 - 1.07) | 3.30E-01 | 0.95 (0.85 - 1.06) | 6.27E-02 | 1.23 (0.99 - 1.53) | 4.71E-02 | 0.84 (0.71 – 1.00) | 3.70E-01 | 0.94 (0.83 - 1.07) |
| 2 | rs840947 | A | 7.16E-01 | 0.98 (0.89 - 1.09) | 6.73E-01 | 0.98 (0.88 - 1.09) | 2.27E-01 | 0.87 (0.70 - 1.09) | 5.46E-01 | 1.05 (0.89 - 1.24) | 9.83E-01 | 1.00 (0.89 - 1.13) |
| 2 | rs1430261 | G | 9.24E-01 | 0.99 (0.84 - 1.17) | 7.15E-01 | 1.03 (0.87 - 1.22) | 5.43E-01 | 0.89 (0.62 - 1.28) | 7.53E-01 | 0.96 (0.73 - 1.25) | 6.25E-01 | 1.05 (0.86 - 1.27) |
| 2 | rs13020362 | G | 2.72E-03 | 0.84 (0.74 - 0.94) | 5.60E-03 | 0.85 (0.75 - 0.95) | 4.43E-02 | 0.78 (0.61 - 0.99) | 4.97E-01 | 0.94 (0.78 - 1.13) | 2.71E-02 | 0.86 (0.75 - 0.98) |
| 3 | rs4855535 | G | 5.97E-01 | 1.04 (0.89 - 1.22) | 3.30E-01 | 1.08 (0.93 - 1.26) | 9.99E-01 | 1.00 (0.73 - 1.37) | 2.54E-01 | 1.15 (0.91 - 1.45) | 8.97E-01 | 1.01 (0.84 - 1.22) |
| 3 | rs4677602 | T | 5.50E-01 | 1.03 (0.93 - 1.15) | 9.01E-01 | 1.01 (0.91 - 1.12) | 2.08E-01 | 1.15 (0.93 - 1.42) | 1.37E-01 | 0.88 (0.74 - 1.04) | 4.87E-01 | 1.04 (0.92 - 1.18) |
| 3 | rs7644066 | G | 3.59E-01 | 1.05 (0.95 - 1.17) | 3.38E-01 | 1.06 (0.95 - 1.18) | 3.03E-01 | 1.12 (0.90 - 1.38) | 5.52E-01 | 0.95 (0.80 - 1.12) | 2.24E-01 | 1.08 (0.95 - 1.23) |
| 3 | rs528581 | C | 1.35E-05 | 1.26 (1.13 - 1.39) | 1.48E-05 | 1.26 (1.13 - 1.40) | 1.26E-02 | 1.31 (1.06 - 1.62) | 7.64E-05 | 1.39 (1.18 - 1.63) | 2.67E-02 | 1.15 (1.02 - 1.30) |
| 3 | rs4680035 | G | 1.19E-02 | 0.88 (0.79 - 0.97) | 4.52E-03 | 0.86 (0.78 - 0.95) | 2.81E-02 | 0.79 (0.63 - 0.97) | 3.96E-02 | 0.84 (0.72 - 0.99) | 2.11E-01 | 0.93 (0.82 - 1.05) |
| 4 | rs2712136 | G | 6.36E-01 | 0.98 (0.88 - 1.08) | 4.19E-01 | 0.96 (0.87 - 1.06) | 9.86E-01 | 1.00 (0.81 - 1.22) | 4.71E-01 | 0.94 (0.81 - 1.10) | 3.78E-01 | 0.95 (0.84 - 1.07) |
| 5 | rs16882931 | T | 3.57E-01 | 0.92 (0.76 - 1.10) | 3.61E-01 | 0.92 (0.76 - 1.10) | 2.93E-01 | 0.81 (0.54 - 1.2) | 2.82E-01 | 0.85 (0.63 - 1.15) | 9.82E-01 | 1.00 (0.81 - 1.24) |
| 5 | rs1115728 | C | 3.50E-01 | 0.95 (0.86 - 1.06) | 4.98E-01 | 0.96 (0.87 - 1.07) | 8.44E-01 | 1.02 (0.83 - 1.26) | 6.54E-01 | 0.96 (0.82 - 1.13) | 3.84E-01 | 0.95 (0.84 - 1.07) |
| 5 | rs11744200 | C | 5.14E-01 | 0.95 (0.80 - 1.12) | 7.18E-01 | 0.97 (0.82 - 1.15) | 3.51E-01 | 1.16 (0.85 - 1.58) | 4.48E-01 | 0.90 (0.69 - 1.18) | 3.45E-01 | 0.90 (0.74 - 1.11) |
| 6 | rs707824 | T | 1.78E-03 | 1.26 (1.09 - 1.45) | 3.34E-04 | 1.31 (1.13 - 1.50) | 1.93E-01 | 1.22 (0.90 - 1.64) | 1.85E-03 | 1.41 (1.14 - 1.76) | 1.65E-02 | 1.22 (1.04 - 1.44) |
| 6 | rs6459451 | C | 3.01E-01 | 1.06 (0.95 - 1.19) | 1.24E-01 | 1.10 (0.98 - 1.23) | 6.98E-02 | 1.24 (0.98 - 1.57) | 3.79E-01 | 1.09 (0.90 - 1.31) | 5.42E-01 | 1.04 (0.91 - 1.20) |
| 6 | rs13199524 | T | 3.05E-02 | 0.82 (0.69 - 0.98) | 4.02E-02 | 0.83 (0.69 - 0.99) | 3.41E-01 | 1.17 (0.84 - 1.63) | 9.22E-04 | 0.57 (0.41 - 0.79) | 1.14E-01 | 0.84 (0.68 - 1.04) |
| 6 | rs9268402 | A | 1.20E-02 | 0.88 (0.79 - 0.97) | 3.93E-02 | 0.89 (0.80 - 0.99) | 1.48E-01 | 1.17 (0.95 - 1.43) | 8.31E-02 | 0.87 (0.74 - 1.02) | 2.34E-04 | 0.79 (0.69 - 0.89) |
| 6 | rs9391858 | G | 5.37E-02 | 0.84 (0.70 – 1.00) | 1.83E-01 | 0.88 (0.74 - 1.06) | 2.22E-01 | 1.22 (0.89 - 1.68) | 1.97E-01 | 0.82 (0.61 - 1.11) | 4.78E-03 | 0.72 (0.58 - 0.91) |
| 6 | rs9268853 | C | 4.32E-01 | 1.04 (0.94 - 1.16) | 3.96E-01 | 1.05 (0.94 - 1.16) | 1.24E-03 | 1.42 (1.15 - 1.75) | 9.70E-01 | 1.00 (0.84 - 1.18) | 3.16E-01 | 0.94 (0.83 - 1.06) |
| 6 | rs4530903 | T | 5.79E-04 | 1.29 (1.12 - 1.49) | 3.48E-04 | 1.31 (1.13 - 1.51) | 1.01E-05 | 1.83 (1.40 - 2.38) | 5.36E-04 | 1.46 (1.18 - 1.82) | 7.56E-01 | 1.03 (0.86 - 1.23) |
| 6 | rs2647046 | A | 5.46E-03 | 0.85 (0.76 - 0.95) | 1.38E-02 | 0.86 (0.76 - 0.97) | 1.21E-03 | 0.67 (0.52 - 0.85) | 3.09E-01 | 0.91 (0.76 - 1.09) | 1.21E-01 | 0.89 (0.78 - 1.03) |
| 6 | rs9275572 | A | 9.05E-04 | 0.83 (0.74 - 0.93) | 2.82E-03 | 0.84 (0.74 - 0.94) | 1.64E-03 | 0.68 (0.54 - 0.87) | 2.06E-01 | 0.89 (0.75 - 1.07) | 3.68E-02 | 0.86 (0.75 - 0.99) |
| 6 | rs9276490 | A | 9.29E-03 | 0.87 (0.78 - 0.97) | 3.06E-02 | 0.89 (0.80 - 0.99) | 9.13E-02 | 0.83 (0.67 - 1.03) | 9.47E-02 | 0.87 (0.73 - 1.03) | 6.77E-02 | 0.89 (0.78 - 1.01) |
| 6 | rs7453920 | A | 4.98E-03 | 0.86 (0.77 - 0.95) | 1.18E-02 | 0.87 (0.77 - 0.97) | 4.35E-02 | 0.79 (0.64 - 0.99) | 8.13E-02 | 0.86 (0.72 - 1.02) | 5.24E-02 | 0.88 (0.77 – 1.00) |
| 6 | rs2621416 | G | 2.75E-01 | 1.06 (0.95 - 1.19) | 9.12E-02 | 1.09 (0.98 - 1.24) | 1.17E-02 | 1.31 (1.07 - 1.66) | 9.27E-02 | 1.15 (0.98 - 1.39) | 5.67E-01 | 0.96 (0.84 - 1.10) |
| 6 | rs9446171 | G | 2.07E-01 | 1.08 (0.96 - 1.21) | 3.37E-01 | 1.06 (0.94 - 1.19) | 7.17E-01 | 1.05 (0.82 - 1.34) | 5.83E-01 | 1.05 (0.88 - 1.26) | 4.00E-01 | 1.06 (0.93 - 1.21) |
| 6 | rs1273725 | A | 5.32E-01 | 0.95 (0.81 - 1.11) | 4.27E-01 | 0.94 (0.80 - 1.10) | 2.68E-01 | 0.82 (0.57 - 1.17) | 1.13E-01 | 0.81 (0.62 - 1.05) | 3.66E-01 | 1.09 (0.91 - 1.30) |
| 7 | rs13233227 | C | 9.46E-01 | 1.00 (0.90 - 1.11) | 3.02E-01 | 0.94 (0.85 - 1.05) | 2.13E-01 | 0.87 (0.69 - 1.09) | 8.85E-01 | 0.99 (0.83 - 1.17) | 8.17E-01 | 0.99 (0.87 - 1.12) |
| 7 | rs1501409 | T | 1.10E-01 | 0.90 (0.79 - 1.02) | 5.18E-01 | 0.96 (0.84 - 1.09) | 6.57E-01 | 0.94 (0.72 - 1.23) | 4.59E-01 | 0.92 (0.75 - 1.14) | 4.30E-01 | 0.94 (0.81 - 1.09) |
| 7 | rs281880 | A | 1.07E-01 | 1.14 (0.97 - 1.35) | 9.55E-02 | 1.16 (0.97 - 1.37) | 7.28E-02 | 1.35 (0.97 - 1.88) | 2.03E-01 | 1.18 (0.91 - 1.52) | 4.81E-01 | 1.08 (0.87 - 1.33) |
| 8 | rs1512384 | T | 6.66E-02 | 1.19 (0.99 - 1.43) | 6.01E-02 | 1.20 (0.99 - 1.45) | 8.53E-01 | 0.96 (0.63 - 1.47) | 6.53E-01 | 1.07 (0.79 - 1.46) | 1.64E-02 | 1.30 (1.05 - 1.60) |
| 9 | rs700983 | T | 6.74E-01 | 0.97 (0.87 - 1.10) | 3.06E-01 | 0.94 (0.83 - 1.06) | 8.92E-01 | 0.98 (0.77 - 1.26) | 4.93E-01 | 0.94 (0.77 - 1.13) | 6.62E-01 | 0.97 (0.84 - 1.12) |
| 9 | rs7026635 | G | 9.29E-01 | 0.99 (0.88 - 1.12) | 9.89E-01 | 1.00 (0.88 - 1.13) | 5.69E-01 | 1.08 (0.84 - 1.38) | 9.89E-01 | 1.00 (0.83 - 1.21) | 9.63E-01 | 1.00 (0.86 - 1.15) |
| 10 | rs12244831 | C | 8.40E-01 | 0.99 (0.88 - 1.11) | 9.82E-01 | 1.00 (0.89 - 1.13) | 2.41E-01 | 0.86 (0.66 - 1.11) | 8.13E-01 | 1.02 (0.85 - 1.23) | 9.71E-01 | 1.00 (0.87 - 1.15) |
| 10 | rs11254308 | T | 4.79E-01 | 1.04 (0.94 - 1.15) | 2.34E-01 | 1.07 (0.96 - 1.19) | 9.94E-01 | 1.00 (0.80 - 1.24) | 4.16E-01 | 1.07 (0.91 - 1.26) | 4.09E-01 | 1.05 (0.93 - 1.19) |
| 11 | rs12289961 | T | 8.29E-04 | 1.22 (1.09 - 1.39) | 1.74E-03 | 1.21 (1.08 - 1.38) | 6.75E-02 | 1.26 (0.98 - 1.60) | 1.97E-02 | 1.25 (1.04 - 1.51) | 1.64E-02 | 1.19 (1.03 - 1.37) |
| 11 | rs948562 | G | 1.33E-03 | 1.23 (1.09 - 1.41) | 2.54E-03 | 1.22 (1.07 - 1.40) | 3.19E-01 | 1.13 (0.88 - 1.50) | 3.24E-02 | 1.24 (1.02 - 1.53) | 1.00E-02 | 1.23 (1.05 - 1.42) |
| 11 | rs11212484 | A | 2.25E-01 | 0.92 (0.80 - 1.05) | 4.91E-01 | 0.95 (0.83 - 1.09) | 9.04E-01 | 0.98 (0.75 - 1.30) | 6.96E-01 | 0.96 (0.77 - 1.19) | 6.16E-01 | 0.96 (0.82 - 1.12) |
| 12 | rs10843211 | G | 2.23E-01 | 0.93 (0.83 - 1.05) | 2.49E-01 | 0.93 (0.83 - 1.05) | 1.19E-01 | 0.82 (0.64 - 1.05) | 8.35E-01 | 0.98 (0.82 - 1.18) | 5.24E-01 | 0.96 (0.83 - 1.10) |
| 13 | rs9561965 | G | 1.44E-01 | 1.13 (0.96 - 1.33) | 2.70E-01 | 1.10 (0.93 - 1.29) | 6.77E-02 | 1.35 (0.98 - 1.87) | 7.45E-02 | 0.77 (0.58 - 1.03) | 2.16E-02 | 1.24 (1.03 - 1.49) |
| 14 | rs17308862 | T | 1.95E-01 | 1.08 (0.96 - 1.22) | 1.16E-01 | 1.10 (0.98 - 1.24) | 2.15E-01 | 1.17 (0.91 - 1.49) | 6.51E-01 | 1.05 (0.86 - 1.27) | 3.97E-01 | 1.06 (0.92 - 1.22) |
| 14 | rs1241129 | G | 9.64E-01 | 1.00 (0.86 - 1.15) | 6.17E-01 | 1.04 (0.90 - 1.20) | 4.94E-01 | 1.11 (0.83 - 1.47) | 5.59E-01 | 0.93 (0.74 - 1.18) | 9.01E-01 | 1.01 (0.86 - 1.19) |
| 14 | rs2356911 | G | 7.10E-01 | 1.02 (0.92 - 1.13) | 6.25E-01 | 1.03 (0.92 - 1.14) | 8.81E-01 | 0.98 (0.79 - 1.22) | 8.88E-01 | 0.99 (0.84 - 1.16) | 2.64E-01 | 1.07 (0.95 - 1.21) |
| 14 | rs10133918 | T | 3.82E-01 | 1.05 (0.94 - 1.16) | 4.17E-01 | 1.04 (0.94 - 1.16) | 9.81E-01 | 1.00 (0.81 - 1.23) | 8.45E-01 | 0.98 (0.84 - 1.16) | 7.87E-02 | 1.11 (0.99 - 1.26) |
| 16 | rs1117412 | C | 2.92E-01 | 1.06 (0.95 - 1.17) | 5.27E-01 | 1.03 (0.93 - 1.15) | 2.87E-01 | 0.89 (0.72 - 1.10) | 6.32E-01 | 1.04 (0.89 - 1.22) | 1.59E-01 | 1.09 (0.97 - 1.23) |
| 17 | rs9303035 | G | 5.08E-01 | 0.96 (0.86 - 1.08) | 9.35E-01 | 1.01 (0.89 - 1.13) | 6.46E-01 | 1.06 (0.83 - 1.34) | 8.14E-01 | 0.98 (0.81 - 1.18) | 6.90E-01 | 0.97 (0.85 - 1.12) |
| 21 | rs235385 | C | 9.78E-02 | 1.09 (0.98 - 1.21) | 1.30E-01 | 1.09 (0.98 - 1.21) | 1.84E-01 | 1.16 (0.93 - 1.44) | 5.05E-01 | 1.06 (0.90 - 1.25) | 1.84E-01 | 1.09 (0.96 - 1.23) |

| **Replication Phase** | | | **Lym** | | **Nhl** | | **FL** | | **Dlbcl** | | **NFD** | |
| --- | --- | --- | --- | --- | --- | --- | --- | --- | --- | --- | --- | --- |
| **Chr** | **SNP** | **RA** | **OR (CI)** | **P** | **OR (CI)** | **P** | **OR (CI)** | **P** | **OR (CI)** | **P** | **OR (CI)** | **P** |
| rs11581364 | T | 0.95 (0.86 - 1.05) | 3.42E-01 | 0.95 (0.86 - 1.06) | 0.3492 | 1.23 (0.99 - 1.53) | 0.06273 | 0.84 (0.71 - 1) | 0.04714 | 0.94 (0.83 - 1.07) | 0.3696 |  |
| 1 | rs2762682 | T | 0.93 (0.83 - 1.03) | 1.72E-01 | 0.93 (0.83 - 1.05) | 0.2335 | 0.92 (0.72 - 1.16) | 0.4672 | 0.92 (0.77 - 1.1) | 0.373 | 0.93 (0.81 - 1.07) | 0.2995 |
| 1 | rs2985340 | C | 0.92 (0.83 - 1.03) | 1.55E-01 | 0.93 (0.83 - 1.04) | 0.2095 | 0.92 (0.73 - 1.17) | 0.5109 | 0.92 (0.77 - 1.1) | 0.365 | 0.92 (0.81 - 1.06) | 0.2504 |
| 1 | rs3767635 | G | 0.97 (0.88 - 1.07) | 5.61E-01 | 0.97 (0.87 - 1.08) | 0.5986 | 1.22 (0.98 - 1.51) | 0.07422 | 0.86 (0.72 - 1.02) | 0.08086 | 0.97 (0.86 - 1.1) | 0.6457 |
| 2 | rs13020362 | G | 0.87 (0.78 - 0.97) | 1.45E-02 | 0.85 (0.76 - 0.96) | 0.006785 | 0.79 (0.62 - 1) | 0.0517 | 0.94 (0.78 - 1.13) | 0.4966 | 0.86 (0.75 - 0.98) | 0.02709 |
| 2 | rs1430261 | G | 1.00 (0.85 - 1.17) | 9.92E-01 | 1.03 (0.87 - 1.22) | 0.751 | 0.87 (0.61 - 1.25) | 0.4605 | 0.96 (0.73 - 1.25) | 0.7528 | 1.05 (0.86 - 1.27) | 0.625 |
| 2 | rs840947 | A | 0.99 (0.89 - 1.09) | 7.83E-01 | 0.98 (0.88 - 1.09) | 0.694 | 0.86 (0.69 - 1.07) | 0.1876 | 1.05 (0.89 - 1.24) | 0.5459 | 1.00 (0.89 - 1.13) | 0.983 |
| 3 | rs4677602 | T | 1.01 (0.91 - 1.12) | 8.19E-01 | 1.01 (0.9 - 1.12) | 0.9075 | 1.13 (0.91 - 1.4) | 0.2568 | 0.88 (0.74 - 1.04) | 0.1367 | 1.04 (0.92 - 1.18) | 0.4872 |
| 3 | rs4680035 | G | 0.88 (0.8 - 0.97) | 1.05E-02 | 0.86 (0.78 - 0.95) | 0.004453 | 0.79 (0.63 - 0.97) | 0.02732 | 0.84 (0.72 - 0.99) | 0.03955 | 0.93 (0.82 - 1.05) | 0.2109 |
| 3 | rs4855535 | G | 1.05 (0.9 - 1.21) | 5.56E-01 | 1.09 (0.93 - 1.27) | 0.2967 | 1.01 (0.74 - 1.38) | 0.9654 | 1.15 (0.91 - 1.45) | 0.2535 | 1.01 (0.84 - 1.22) | 0.8971 |
| 3 | rs528581 | C | 1.24 (1.12 - 1.37) | 2.40E-05 | 1.26 (1.14 - 1.4) | 0.00001317 | 1.32 (1.07 - 1.64) | 0.01003 | 1.39 (1.18 - 1.63) | 0.00007637 | 1.15 (1.02 - 1.3) | 0.02665 |
| 3 | rs7644066 | G | 1.05 (0.95 - 1.17) | 3.59E-01 | 1.06 (0.95 - 1.18) | 0.3143 | 1.12 (0.9 - 1.38) | 0.3034 | 0.95 (0.8 - 1.12) | 0.552 | 1.08 (0.95 - 1.23) | 0.2235 |
| 4 | rs2712136 | G | 0.96 (0.87 - 1.05) | 3.50E-01 | 0.96 (0.87 - 1.06) | 0.4138 | 1.00 (0.81 - 1.22) | 0.9801 | 0.94 (0.81 - 1.1) | 0.4709 | 0.95 (0.84 - 1.07) | 0.3782 |
| 5 | rs1115728 | C | 0.97 (0.88 - 1.07) | 5.29E-01 | 0.97 (0.87 - 1.07) | 0.5103 | 1.02 (0.82 - 1.26) | 0.8601 | 0.96 (0.82 - 1.13) | 0.6535 | 0.95 (0.84 - 1.07) | 0.3839 |
| 5 | rs11744200 | C | 0.95 (0.8 - 1.12) | 5.14E-01 | 0.97 (0.82 - 1.15) | 0.7113 | 1.16 (0.85 - 1.58) | 0.3513 | 0.9 (0.69 - 1.18) | 0.4475 | 0.9 (0.74 - 1.11) | 0.345 |
| 5 | rs16882931 | T | 0.92 (0.77 - 1.1) | 3.50E-01 | 0.92 (0.76 - 1.11) | 0.3677 | 0.81 (0.55 - 1.21) | 0.3073 | 0.85 (0.63 - 1.15) | 0.2823 | 1.00 (0.81 - 1.24) | 0.9818 |
| 6 | rs7453920 | A | 0.86 (0.77 - 0.95) | 4.98E-03 | 0.87 (0.78 - 0.97) | 0.01229 | 0.79 (0.64 - 0.99) | 0.04359 | 0.86 (0.72 - 1.02) | 0.08132 | 0.88 (0.77 - 1) | 0.05239 |
| 6 | rs1273725 | A | 0.96 (0.83 - 1.12) | 6.26E-01 | 0.94 (0.8 - 1.1) | 0.4322 | 0.82 (0.58 - 1.17) | 0.2811 | 0.81 (0.62 - 1.05) | 0.1132 | 1.09 (0.91 - 1.3) | 0.3662 |
| 6 | rs13199524 | T | 0.82 (0.69 - 0.97) | 2.15E-02 | 0.82 (0.69 - 0.99) | 0.03555 | 1.15 (0.83 - 1.6) | 0.408 | 0.57 (0.41 - 0.79) | 0.0009222 | 0.84 (0.68 - 1.04) | 0.1138 |
| 6 | rs2621416 | G | 1.07 (0.95 - 1.19) | 2.62E-01 | 1.10 (0.98 - 1.23) | 0.1018 | 1.33 (1.06 - 1.66) | 0.0133 | 1.16 (0.98 - 1.39) | 0.09272 | 0.96 (0.84 - 1.1) | 0.5671 |
| 6 | rs2647046 | A | 0.85 (0.76 - 0.95) | 5.45E-03 | 0.86 (0.77 - 0.97) | 0.01666 | 0.67 (0.52 - 0.85) | 0.001207 | 0.91 (0.76 - 1.09) | 0.3099 | 0.89 (0.78 - 1.03) | 0.1213 |
| 6 | rs4530903 | T | 1.26 (1.09 - 1.45) | 5.79E-04 | 1.29 (1.12 - 1.49) | 0.0003485 | 1.31 (1.13 – 1.51) | 0.000008247 | 1.46 (1.18 - 1.82) | 0.000536 | 1.03 (0.86 - 1.23) | 0.7557 |
| 6 | rs6459451 | C | 1.09 (0.97 - 1.22) | 1.47E-01 | 1.09 (0.97 - 1.23) | 0.1357 | 1.25 (0.99 - 1.58) | 0.06125 | 1.09 (0.9 - 1.31) | 0.3787 | 1.04 (0.91 - 1.2) | 0.5422 |
| 6 | rs707824 | T | 1.26 (1.1 - 1.45) | 1.78E-03 | 1.3 (1.12 - 1.5) | 0.0003345 | 1.2 (0.89 - 1.62) | 0.193 | 1.41 (1.14 - 1.76) | 0.001852 | 1.22 (1.04 - 1.44) | 0.01648 |
| 6 | rs9268402 | A | 0.88 (0.79 - 0.97) | 1.20E-02 | 0.89 (0.8 - 0.99) | 0.03665 | 1.17 (0.95 - 1.43) | 0.1478 | 0.87 (0.74 - 1.02) | 0.08308 | 0.79 (0.69 - 0.89) | 0.0002335 |
| 6 | rs9268853 | C | 1.04 (0.94 - 1.16) | 4.31E-01 | 1.04 (0.94 - 1.16) | 0.3963 | 1.42 (1.13 - 1.73) | 0.001245 | 1.00 (0.84 - 1.18) | 0.9701 | 0.94 (0.83 - 1.06) | 0.3157 |
| 6 | rs9275572 | A | 0.83 (0.74 - 0.93) | 9.05E-04 | 0.84 (0.75 - 0.94) | 0.003475 | 0.68 (0.54 - 0.87) | 0.001637 | 0.89 (0.75 - 1.07) | 0.2057 | 0.86 (0.75 - 0.99) | 0.03677 |
| 6 | rs9391858 | G | 0.83 (0.7 - 0.99) | 4.04E-02 | 0.88 (0.73 - 1.06) | 0.1717 | 1.2 (0.87 - 1.66) | 0.2638 | 0.82 (0.61 - 1.11) | 0.197 | 0.72 (0.58 - 0.91) | 0.004778 |
| 6 | rs9446171 | G | 1.06 (0.95 - 1.19) | 2.93E-01 | 1.06 (0.94 - 1.19) | 0.3319 | 1.04 (0.81 - 1.33) | 0.7635 | 1.05 (0.88 - 1.26) | 0.5833 | 1.06 (0.93 - 1.21) | 0.3996 |
| 6 | rs9276490 | A | 0.87 (0.78 - 0.97) | 9.29E-03 | 0.89 (0.8 - 0.99) | 0.03164 | 0.83 (0.67 - 1.03) | 0.09132 | 0.87 (0.73 - 1.03) | 0.09474 | 0.89 (0.78 - 1.01) | 0.06767 |
| 7 | rs13233227 | C | 0.97 (0.88 - 1.08) | 5.82E-01 | 0.94 (0.85 - 1.05) | 0.281 | 0.86 (0.69 - 1.08) | 0.2004 | 0.99 (0.83 - 1.17) | 0.8852 | 0.99 (0.87 - 1.12) | 0.8174 |
| 7 | rs1501409 | T | 0.94 (0.83 - 1.06) | 3.05E-01 | 0.96 (0.84 - 1.09) | 0.5303 | 0.95 (0.72 - 1.24) | 0.6906 | 0.92 (0.75 - 1.14) | 0.4589 | 0.94 (0.81 - 1.09) | 0.4296 |
| 7 | rs281880 | A | 1.14 (0.97 - 1.35) | 1.07E-01 | 1.16 (0.98 - 1.38) | 0.08674 | 1.35 (0.97 - 1.88) | 0.07284 | 1.18 (0.91 - 1.52) | 0.2028 | 1.08 (0.87 - 1.33) | 0.4811 |
| 8 | rs1512384 | T | 1.19 (0.99 - 1.42) | 6.21E-02 | 1.2 (0.99 - 1.45) | 0.05975 | 0.96 (0.63 - 1.48) | 0.8685 | 1.07 (0.79 - 1.46) | 0.6531 | 1.3 (1.05 - 1.6) | 0.01638 |
| 9 | rs700983 | T | 0.96 (0.86 - 1.08) | 5.20E-01 | 0.94 (0.83 - 1.06) | 0.2819 | 0.98 (0.76 - 1.25) | 0.8498 | 0.94 (0.77 - 1.13) | 0.4932 | 0.97 (0.84 - 1.12) | 0.6618 |
| 9 | rs7026635 | G | 1.00 (0.89 - 1.13) | 9.43E-01 | 1.00 (0.88 - 1.13) | 0.9955 | 1.07 (0.83 - 1.37) | 0.608 | 1.00 (0.83 - 1.21) | 0.9893 | 1.00 (0.86 - 1.15) | 0.9627 |
| 10 | rs11254308 | T | 1.04 (0.94 - 1.15) | 4.25E-01 | 1.07 (0.96 - 1.19) | 0.2349 | 1.01 (0.81 - 1.26) | 0.9435 | 1.07 (0.91 - 1.26) | 0.4164 | 1.05 (0.93 - 1.19) | 0.4093 |
| 10 | rs12244831 | C | 0.98 (0.87 - 1.1) | 7.41E-01 | 1.00 (0.89 - 1.13) | 0.9836 | 0.86 (0.67 - 1.12) | 0.2617 | 1.02 (0.85 - 1.23) | 0.8133 | 1.00 (0.87 - 1.15) | 0.971 |
| 11 | rs11212484 | A | 0.95 (0.84 - 1.09) | 4.72E-01 | 0.95 (0.83 - 1.09) | 0.4663 | 0.97 (0.74 - 1.28) | 0.8453 | 0.96 (0.77 - 1.19) | 0.6955 | 0.96 (0.82 - 1.12) | 0.6155 |
| 11 | rs12289961 | T | 1.21 (1.08 - 1.36) | 1.40E-03 | 1.22 (1.08 - 1.38) | 0.001663 | 1.26 (0.99 - 1.61) | 0.06048 | 1.25 (1.04 - 1.51) | 0.01966 | 1.19 (1.03 - 1.37) | 0.01644 |
| 11 | rs948562 | G | 1.21 (1.07 - 1.38) | 2.72E-03 | 1.23 (1.07 - 1.4) | 0.002564 | 1.15 (0.88 - 1.5) | 0.3193 | 1.25 (1.02 - 1.53) | 0.03249 | 1.22 (1.05 - 1.42) | 0.01004 |
| 12 | rs10843211 | G | 0.94 (0.84 - 1.05) | 2.68E-01 | 0.93 (0.83 - 1.05) | 0.2417 | 0.8 (0.62 - 1.03) | 0.08516 | 0.98 (0.82 - 1.18) | 0.8353 | 0.96 (0.83 - 1.1) | 0.5238 |
| 13 | rs9561965 | G | 1.12 (0.96 - 1.31) | 1.62E-01 | 1.1 (0.93 - 1.29) | 0.271 | 1.36 (0.98 - 1.88) | 0.06416 | 0.77 (0.58 - 1.03) | 0.0745 | 1.24 (1.03 - 1.49) | 0.02157 |
| 14 | rs10133918 | T | 1.06 (0.96 - 1.17) | 2.53E-01 | 1.04 (0.94 - 1.15) | 0.4569 | 0.99 (0.8 - 1.22) | 0.8947 | 0.98 (0.84 - 1.16) | 0.8447 | 1.11 (0.99 - 1.26) | 0.07873 |
| 14 | rs1241129 | G | 1.01 (0.88 - 1.16) | 9.00E-01 | 1.03 (0.89 - 1.19) | 0.6578 | 1.09 (0.82 - 1.46) | 0.5527 | 0.93 (0.74 - 1.18) | 0.5585 | 1.01 (0.86 - 1.19) | 0.9012 |
| 14 | rs17308862 | T | 1.07 (0.95 - 1.2) | 2.64E-01 | 1.1 (0.97 - 1.24) | 0.1358 | 1.14 (0.9 - 1.46) | 0.282 | 1.05 (0.86 - 1.27) | 0.6514 | 1.06 (0.92 - 1.22) | 0.3969 |
| 14 | rs2356911 | G | 1.03 (0.93 - 1.14) | 5.26E-01 | 1.02 (0.92 - 1.14) | 0.6738 | 0.97 (0.79 - 1.2) | 0.7973 | 0.99 (0.84 - 1.16) | 0.8883 | 1.07 (0.95 - 1.21) | 0.2642 |
| 16 | rs1117412 | C | 1.05 (0.95 - 1.16) | 3.40E-01 | 1.03 (0.93 - 1.14) | 0.5508 | 0.89 (0.72 - 1.1) | 0.2835 | 1.04 (0.89 - 1.22) | 0.6321 | 1.09 (0.97 - 1.23) | 0.1587 |
| 17 | rs9303035 | G | 0.98 (0.88 - 1.1) | 7.58E-01 | 1.00 (0.89 - 1.13) | 0.9769 | 1.05 (0.83 - 1.34) | 0.6826 | 0.98 (0.81 - 1.18) | 0.8139 | 0.97 (0.85 - 1.12) | 0.6899 |
| 21 | rs235385 | C | 1.08 (0.97 - 1.19) | 1.54E-01 | 1.09 (0.98 - 1.21) | 0.1316 | 1.16 (0.93 - 1.43) | 0.1898 | 1.06 (0.9 - 1.25) | 0.5049 | 1.09 (0.96 - 1.23) | 0.1835 |

LYM=All lymphoma, NHL=Non-Hodgkin’s lymphoma, FL=follicular lymphoma and DLBCL=diffuse large B cell lymphoma, NFD=Non-follicular and non-diffuse large B cell subtypes. SNP=rsID of the single nucleotide polymorphism, Chr=chromosome, RA=risk allele, OR (CI)=odds-ratio (Confidence interval, lower-upper).
