# Supplementary material for: Susceptibility Loci Associated with Specific and Shared Subtypes of Lymphoid Malignancies
Source: PLoS Genet. 2013 Jan 17;9(1):e1003220. doi: 10.1371/journal.pgen.1003220 (PMC3547842; doi:10.1371/journal.pgen.1003220)
Supplement: Table S3 — Correlation and recombination rate of SNPs associated with the HLA and 11q12.1 regions in HapMap. (DOCX) [file pgen.1003220.s010.docx]

**Table S3: Correlation and recombination rate of SNPs associated with the HLA and 11q12.1 regions in HapMap.**

| ***Chr*** | ***BP*** | ***SNP1*** | ***SNP2*** | ***Distance*** | ***r^2^*** | ***D'*** | ***Ɵ(cM/Mb)*** | **Distance (cM)** |
| --- | --- | --- | --- | --- | --- | --- | --- | --- |
| chr6 | 32537621 | rs7453920 | rs9268853 | 300369 | 0.01 | 0.15 | 0.047 | 0.225 |
| chr6 | 32537621 | rs2621416 | rs9268853 | 312225 | 0.01 | 0.15 | 0.047 | 0.253 |
| chr6 | 32826659 | rs9268853 | rs9276490 | 289038 | 0.02 | 0.15 | 0.004 | 0.222 |
| chr6 | 32537621 | rs4530903 | rs9268853 | 152246 | 0.07 | 0.90 | 0.047 | 0.040 |
| chr6 | 32689867 | rs2647046 | rs4530903 | 86447 | 0.07 | 1.00 | 7.083 | 0.077 |
| chr6 | 32537621 | rs7755224 | rs9268853 | 222674 | 0.08 | 1.00 | 0.047 | 0.087 |
| chr6 | 32537621 | rs10484561 | rs9268853 | 235777 | 0.08 | 1.00 | 0.047 | 0.109 |
| chr6 | 32772436 | rs10484561 | rs2647012 | 962 | 0.09 | 1.00 | 1.430 | 0.002 |
| chr6 | 32776314 | rs10484561 | rs2647046 | 2916 | 0.09 | 1.00 | 5.829 | 0.008 |
| chr6 | 32772436 | rs7755224 | rs2647012 | 12141 | 0.09 | 1.00 | 1.430 | 0.020 |
| chr6 | 32776314 | rs7755224 | rs2647046 | 16019 | 0.09 | 1.00 | 5.829 | 0.029 |
| chr6 | 32826659 | rs10484561 | rs9276490 | 53261 | 0.09 | 0.85 | 0.004 | 0.113 |
| chr6 | 32837990 | rs10484561 | rs7453920 | 64592 | 0.09 | 0.85 | 0.058 | 0.116 |
| chr6 | 32826659 | rs7755224 | rs9276490 | 66364 | 0.09 | 0.85 | 0.004 | 0.135 |
| chr6 | 32837990 | rs7755224 | rs7453920 | 77695 | 0.09 | 0.85 | 0.058 | 0.138 |
| chr6 | 32837990 | rs4530903 | rs7453920 | 148123 | 0.09 | 0.92 | 0.058 | 0.185 |
| chr6 | 32689867 | rs9276490 | rs4530903 | 136792 | 0.10 | 0.92 | 7.083 | 0.182 |
| chr6 | 32689867 | rs2621416 | rs4530903 | 159979 | 0.15 | 0.68 | 7.083 | 0.213 |
| chr6 | 32552176 | rs2621416 | rs12194148 | 297670 | 0.18 | 0.69 | 0.024 | 0.246 |
| chr6 | 32776314 | rs9276490 | rs2647046 | 50345 | 0.19 | 0.54 | 5.829 | 0.105 |
| chr6 | 32837990 | rs2647046 | rs7453920 | 61676 | 0.19 | 0.54 | 0.058 | 0.109 |
| chr6 | 32837990 | rs2621416 | rs7453920 | 11856 | 0.24 | 0.77 | 0.058 | 0.028 |
| chr6 | 32826659 | rs2621416 | rs9276490 | 23187 | 0.24 | 0.77 | 0.004 | 0.031 |
| chr6 | 32776314 | rs2621416 | rs2647046 | 73532 | 0.24 | 0.93 | 5.829 | 0.136 |
| chr6 | 32849846 | rs10484561 | rs2621416 | 76448 | 0.26 | 0.89 | 0.075 | 0.144 |
| chr6 | 32849846 | rs7755224 | rs2621416 | 89551 | 0.26 | 0.89 | 0.075 | 0.166 |
| chr6 | 32537621 | rs2647046 | rs9268853 | 238693 | 0.44 | 1.00 | 0.047 | 0.117 |
| chr6 | 32689867 | rs7755224 | rs4530903 | 70428 | 0.55 | 0.74 | 7.083 | 0.047 |
| chr6 | 32689867 | rs10484561 | rs4530903 | 83531 | 0.55 | 0.74 | 7.083 | 0.069 |
| chr11 | 57816768 | rs948562 | rs12289961 | 287573 | 0.60 | 0.86 | 0.119 | 0.023 |
| chr6 | 32826659 | rs7453920 | rs9276490 | 11331 | 0.98 | 1.00 | 0.004 | 0.003 |
| chr6 | 32760295 | rs10484561 | rs7755224 | 13103 | 1.00 | 1.00 | 1.738 | 0.022 |
