# Supplementary material for: Susceptibility Loci Associated with Specific and Shared Subtypes of Lymphoid Malignancies
Source: PLoS Genet. 2013 Jan 17;9(1):e1003220. doi: 10.1371/journal.pgen.1003220 (PMC3547842; doi:10.1371/journal.pgen.1003220)
Supplement: Table S4 — Sample sizes in Stage-1 and Stage-2. The samples for the phase 1 were collected at MSKCC (N = 860), Dana Farber Cancer Institute (N = 74) and Hadassah Hebrew University, Israel (N = 10). The replication phase was ascertained at MSKCC. (DOCX) [file pgen.1003220.s011.docx]

| **Type/Subtype** | **Stage-1** | **Stage-2** |
| --- | --- | --- |
| Follicular | 275 | 202 |
| DLBCL | 269 | 367 |
| Hodgkin's | 202 | 99 |
| SLL/CLL | 69 | 127 |
| Mantle Cell | 64 | 51 |
| Marginal zone/MALT | 25 | 98 |
| NHL NOS | 13 | 8 |
| Burkitt's | 7 | 11 |
| Waldenstrom's | 6 | 27 |
| Multiple Myeloma | 6 | 168 |
| Anaplastic large cell | 4 | 16 |
| Hairy Cell | 2 | 9 |
| Angioimmunoblastic | 1 | 13 |
| Peripheral T-Cell | 1 | 38 |
| Gastric | 0 | 1 |
| T-lymphoblastic | 0 | 10 |
| **Total** | **944** | **1245** |

**Table S4: Sample Sizes in stage-1 and stage-2.** The samples for the phase 1 were collected at MSKCC (N=860), Dana Farber Cancer Institute (N=74) and Hadassah Hebrew University, Israel (N=10). The replication phase was ascertained at MSKCC.
